# Does 3-Day Course of Oral Amoxycillin Benefit Children of Non-Severe Pneumonia with Wheeze: A Multicentric Randomised Controlled Trial

**DOI:** 10.1371/journal.pone.0001991

**Published:** 2008-04-23

**Authors:** Shally Awasthi, Girdhar Agarwal, Sushil K. Kabra, Sunit Singhi, Madhuri Kulkarni, Vaishali More, Abhimanyu Niswade, Raj Mohan Pillai, Ravi Luke, Neeraj M. Srivastava, Saradha Suresh, Valsan P. Verghese, P. Raghupathy, R. Lodha, Stephen D. Walter

**Affiliations:** 1 King George's Medical University, Lucknow, India; 2 Lucknow University, Lucknow, India; 3 All India Institute of Medical Science, New Delhi, India; 4 Post Graduate Institute of Medical Sciences, Chandigarh, India; 5 Lok Manya Tilak Medical College, Mumbai, India; 6 Government Medical College, Nagpur, India; 7 Government Medical College, Trivandrum, India; 8 Madras Medical College, Chennai, India; 9 Christian Medical College, Vellore, India; 10 McMaster University, Hamilton, Canada; Canadian Agency for Drugs and Technologies in Health, Canada

## Abstract

**Background:**

WHO-defined pneumonias, treated with antibiotics, are responsible for a significant proportion of childhood morbidity and mortality in the developing countries. Since substantial proportion pneumonias have a viral etiology, where children are more likely to present with wheeze, there is a concern that currently antibiotics are being over-prescribed for it. Hence the current trial was conducted with the objective to show the therapeutic equivalence of two treatments (placebo and amoxycillin) for children presenting with non-severe pneumonia with wheeze, who have persistent fast breathing after nebulisation with salbutamol, and have normal chest radiograph.

**Methodology:**

This multi-centric, randomised placebo controlled double blind clinical trial intended to investigate equivalent efficacy of placebo and amoxicillin and was conducted in ambulatory care settings in eight government hospitals in India. Participants were children aged 2–59 months of age, who received either oral amoxycillin (31–54 mg/Kg/day, in three divided doses for three days) or placebo, and standard bronchodilator therapy. Primary outcome was clinical failure on or before day- 4.

**Principal Findings:**

We randomized 836 cases in placebo and 835 in amoxycillin group. Clinical failures occurred in 201 (24.0%) on placebo and 166 (19.9%) on amoxycillin (risk difference 4.2% in favour of antibiotic, 95% CI: 0.2 to 8.1). Adherence for both placebo and amoxycillin was >96% and 98.9% subjects were followed up on day- 4. Clinical failure was associated with (i) placebo treatment (adjusted OR = 1.28, 95% CI: 1.01 to1.62), (ii) excess respiratory rate of >10 breaths per minute (adjusted OR = 1.51, 95% CI: 1.19, 1.92), (iii) vomiting at enrolment (adjusted OR = 1.49, 95% CI: 1.13, 1.96), (iv) history of use of broncho-dilators (adjusted OR = 1.71, 95% CI: 1.30, 2.24) and (v) non-adherence (adjusted OR = 8.06, 95% CI: 4.36, 14.92).

**Conclusions:**

Treating children with non-severe pneumonia and wheeze with a placebo is not equivalent to treatment with oral amoxycillin.

**Trial Registration:**

ClinicalTrials.gov NCT00407394

## Introduction

Acute lower respiratory infections (ALRI) are a leading cause of morbidity and mortality in children under five years of age in developing countries and are responsible for an estimated 2.6 million deaths annually [1]–[3]. Wheezing and reactive airways disease is associated with or contributory to a significant proportion of childhood acute respiratory infections (ARI) [4]–[6]. The current Integrated Management of Childhood Illness (IMCI) algorithm prescribes that children with wheeze and fast breathing presenting to first level health facilities be given antibiotics if they continue to have fast breathing after two doses of bronchodilators [7]. However, an unknown proportion of children managed thus will have viral related wheezing illness like bronchiolitis and asthma rather than pneumonia, where antibiotics may not alter the course of disease. Using IMCI algorithms are likely to result in unnecessary administration of antibiotics as well as inadequate treatment of wheeze [8].

Since our study hypothesis was that when given with an oral bronchodilator, 3-days treatment with either oral amoxycillin or oral placebo would be as effective, in terms of clinical cure on day- 4, we designed a double blind equivalence trial. [9]. The goal of this trial was to assess whether children of non-severe pneumonia with wheeze can be effectively managed without antibiotics and to identify which sub-group of cases do require antibiotics.

## Methods

This was a multi-centric, double blind placebo-controlled randomised trial conducted in outpatient departments of 8 referral hospitals in India. Individual institutional ethics committees' as well as the IndiaClen Institutional Review Board approved the study. It was designed for equivalence of two modes of therapy with a predefined range of equivalence as an interval from −5% to +5%. The trial was registered with the Clinical Trials registry (NCT00407394). The protocol for this trial and supporing CONSORT checklist are available as supporting information; see Checklist S1 and Protocol S1.

Two-stage screening was done (verbal followed by standardised screening) for inclusion and exclusion criteria. Children aged 2–59 months were verbally screened for complaints of cough, rapid respiration, or difficulties in breathing. Excluded were those with signs of WHO defined severe pneumonia or very severe disease[7], other conditions requiring antibiotics therapy, clinically recognized congenital heart disease, chronic systemic disorders, hospitalisation in past 2 weeks, use of antibiotics in previous 2 days, measles within the last month, history of penicillin allergy and prior enrolment in the study. Those who were verbal screening positive and had no exclusion criteria were examined for study entry criteria, which were audible or auscultatory wheeze and WHO defined fast breathing, that is respiratory rate of ≥50 per minute (for age 2–11 months) or ≥40 per minute (for age 12–59 months) [10]. Those who fulfilled the study entry criteria were given a maximum of three doses of salbutamol (400 microgm/dose) by either a nebuliser or metered dose inhaler and their respiratory rate was reassessed. If fast breathing persisted above WHO defined age specific cut-offs, a chest radiograph was taken for diagnosing radiological pneumonia, defined as presence of fluids, parenchymal infiltrates or consolidation [10]. Those with radiological pneumonia were treated according to the standard hospital treatment guidelines. The rest were invited to participate in the study. Those who accepted the invitation were requested to provide informed written parental consent in local language. Local languages used were site specific and were Hindi for Lucknow, Delhi and Chandigarh sites, Marathi for Nagpur and Mumbai sites, Tamil for Chennai and Vellore site and Malayali for Trivandrum site. Children of parents consenting for participation were randomised to receive amoxycillin or placebo and continued with oral salbutamol. Coordinating centre ensured standardised training and quality assurance.

### Follow up

All the participants were followed up on day- 4 and between days 11–14 days. Home visits were done within 24 hours for those who failed to report on the appointed days. Children who failed on therapy, developed adverse reactions or withdrew consent were treated according to standard hospital guidelines.

### Intervention

Participants received either amoxycillin, 31–54 mg/Kg/day, in three divided doses, or placebo, which looked and tasted similar to amoxycillin, in three divided doses orally. In addition all participants also received oral salbutamol, 2.5 ml or 5 ml thrice a day for ages 2–11 months and 12–59 months, respectively (each 5 ml = 2 mg salbutamol). Patients with fever also received paracetemol (10–15 mg/kg/dose) in addition to the study intervention.

### Objectives

The primary objective was to compare the proportions of eligible cases that achieve clinical cure on day- 4 on three-days of treatment with either oral amoxycillin or placebo. Our secondary objective was to compare the proportions that clinically relapsed within the next 11–14 days, as well as identify the determinants, if any, of clinical failure. Secondary laboratory outcome was the proportions positive for respiratory syncycial virus (RSV) in nasopharyngeal aspirate at enrolment.

### Outcome measures

Main outcome measure was clinical cure on day- 4, defined as those who were not assessed to have clinical failure. Clinical failure was defined as either (i) development of WHO-defined severe pneumonia or very severe disease (with or without wheeze) before or on day- 4 assessment, or (ii) oxygen saturation on pulse oximetry <90% before or on day- 4 assessment or (iii) axillary temperature >101 degrees F on day- 4 assessment, or (iv) persistence of WHO-defined non-severe pneumonia on day- 4 assessment, or (v) presence of wheeze on day- 4 assessment. Loss to follow up on day- 4 or withdrawal from the study at any time after recruitment was also considered as therapy failure in the intention-to-treatment (ITT) analysis. Clinical relapse was defined as cases, which were clinically cured on day-4 assessment, but at days-11-14 follow-up showed signs of WHO-defined pneumonia.

### Sample size

This was calculated for therapeutic equivalence of two treatments (placebo and amoxycillin). It was assumed that the overall failure rate would be 17% if the treatments were equivalent [5]. We used δ = 0.05 to be the range of equivalence for the difference in failure rates. To detect this difference using Type I error probability (alpha) of 0.05 and power of 0.9, the required size of each treatment group was 970.

### Randomisation

A professional not associated with clinical care (GGA) generated the randomization scheme at Lucknow. Randomised block sizes of two to four were used to avoid any uneven distribution of patients between two interventions. Unlabelled medicines were placed in serially numbered opaque envelopes by the pharmacy. Doses were provided for a maximum weight of 20 Kg. Extra doses were kept for administration to a child who vomited within 30 minutes of dosing. Health care workers, patients as well as research staff and investigators were blinded.

### Adherence

Adherence was assessed by pill count on day- 4 follow-up. Non-adherence was defined as intake of less than 7 doses.

### Laboratory procedures

A chest radiograph was taken prior to randomisation. Nasopharyngeal aspirate was tested at enrolment for RSV using Directogen^R^ kit (manufactured by Becton Dickinson and Company, Sparks MD 21152, USA).

### Statistical Analysis

Primary analyses were done on ITT basis. Per protocol analysis was also done on subjects with complete follow-up and adherence to treatment. We have used the two one-sided tests (TOST) procedure as well as the confidence interval approach for assessing therapeutic equivalence of two treatments [11]. Baseline and other characteristics were compared between the two regimens. Association of clinical failure with different characteristics of patients was assessed. Chi-squared test was used for testing two-sided hypotheses, whereas Z- test was used to test one-sided hypotheses. A p value of <0.05 was considered to be statistically significant. Crude odds ratios and 95% confidence intervals were computed. The covariates, which were found to be significant or nearly significant (p≤0.10) in univariate analysis, were included to construct a multivariate model for assessing determinants of treatment failure by forward stepwise logistic regression. In the later model, we also investigated the possibility of variables acting as effect modifier for the risk factors. Final model included covariates that were statistically significant. The primary outcome measure was also assessed for each site and the overall odds ratio after stratifying for site was obtained. Mantel-Haenszel statistic for testing independence of clinical failure with interventions, adjusting for site was used. Breslow-Day statistic for testing three-factor interaction of clinical failure, interventions and site was used. Data were analyzed using statistical software “Statistical Package for Social Sciences” (SPSS) 11.0 version.

## Results

### Population

**Figure 1:**
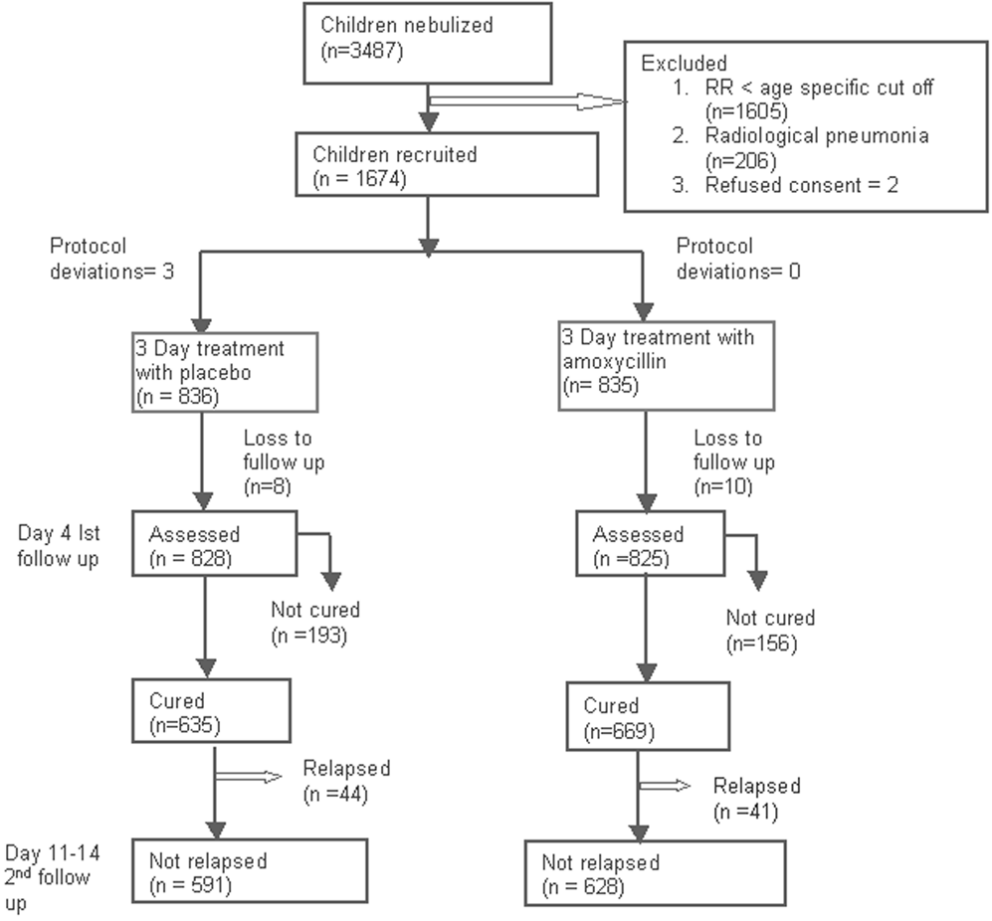
CONSORT Statement for the trial.

We nebulised 3487 children of non-severe pneumonia with wheeze and in 46% (n = 1604), the respiratory rate returned to below age-specific cut-off (Figure 1). From among the remaining, we recruited 1671 patients from February 2004 to July 2006 (Figure 1), 836 in the placebo and 835 in the amoxycillin group. Loss to follow-up was 1% (n = 8) and 1.2% (n = 12) in the placebo and amoxycillin groups by day-4, respectively and 3.6% (n = 30) and 4.6% (n = 38), respectively by days-11-14.

### Baseline characteristics

There were no substantial differences in the baseline characteristics of the treatment groups (Table 1).

**Table 1:** Comparison of Baseline characteristics of patients' pneumonia by treatment type.

CONTINUOUS VARIABLES	Placebo	Amoxycillin
	(n = 836)	(n = 835)
	Mean, SD	Mean, SD
Age (months)	19.5, 14.3	20.0, 14.6
Weight (Kg)	9.1, 2.5	9.2, 2.7
Height (Cm)	76.4, 12.1	76.6, 12.4
Duration of illness (days)	3.9, 1.8	3.8, 1.8
Temperature (°Fahrenheit)	98.9, 0.9	98.9, 0.9
Respiratory Rate (breaths/minute)	52.6, 7.3	52.5, 7.5
Heart Rate (per minute)	115.8, 16.5	115.5, 16.4

CATEGORICAL VARIABLES	Placebo	Amoxycillin
	(n, %	n, %
Female	301, 35.9	300, 35.9
Age 2–11months	334, 39.8	322, 38.6
Weight for height<−3 SD	33, 3.9	33, 4.0
**History of Current Illness**		
Duration of illness> = 3days	651, 77.6	637, 76.3
Fever	468, 55.8	470, 56.3
Vomiting	183, 21.8	164, 19.6
Diarrhoea	8, 1.0	5, 0.6
**History of Allergic disorders in subject**		
Asthma	109, 13.0	107, 12.8
Allergic rhinitis	55, 6.6	54, 6.5
Eczema	6, 0.7	4, 0.5
Urticarial rash	7, 0.8	4, 0.5
Use of Bronchodilator	184, 21.9	183, 21.9
**History of exposure Pollutants**		
Smoker in family	245, 29.2	271, 32.5
Biomass fuel use for cooking	349, 41.6	348, 41.7
LPG use for cooking	531, 63.3	520, 62.3
**Examination**		
Respiratory Rate>10 breaths per minute above cut-off	333, 39.7	340, 40.7
Wheeze (On Auscultation)	704, 83.9	720, 86.2
Wheeze (Audible)	135, 16.1	115, 13.8
**RSV positive** 1	48/778, 6.2	40/780, 5.1

1 For these variables, the results are presented in fractions, as the denominators are different.

*Abbreviations:*

SD: Standard deviation, RSV: Respiratory syncitial virus, LPG: Liquid Petroleum Gas

### Adherence to therapy

Adherence, defined as intake of 7 or more doses, for placebo was 96.1% (791/823) and amoxycillin was 97.8% (801/819) with the difference of −1.7% (95% CI: −3.3 to −0.03). In the placebo and amoxycillin groups 88.1% (675/766) and 90.1% (686/761) took 7 or more doses of oral bronchodilators (difference −2.0%; 95% CI: −5.1 to 1.1).

### Primary and secondary Clinical Outcomes

On ITT analysis, the clinical failure rates were 24.0% (201/836) and 19.9% (166/835) in the placebo and amoxycillin groups, respectively, and the rate difference was 4.2% (95% CI: 0.2 to 8.2) (Table 2). Comparing clinical failures in the two groups on per protocol analysis gave very similar results. Here the confidence interval for the risk difference (p_A_−p_B_) between the two treatments covers at least some points which lie outside the equivalence range (−δ, +δ). Note that δ, the magnitude of difference of clinical importance is taken to be 5.0% here. Now we used TOST approach and tested one-sided hypothesis that the placebo is non-inferior to amoxycillin, i.e. H_0_ : p_A_−p_B_≥δ (in-equivalence) vs H_1_: p_A_−p_B_<δ (equivalence). The corresponding test-statistic is z = −0.41 (p = 0.34) (Table 2). We conclude, therefore, that the hypothesis of “in-equivalence” cannot be disproved. The numbers needed to treat (NNT) to avoid one clinical failure (or get one additional cure) using amoxycillin is 24.

**Table 2:** Comparison of outcome measures in the two treatment groups.

PRIMARY OUTCOME: FAILURE RATE	Placebo	Amoxycillin	Difference,	p value
	m/n, %	m/n, %	95% C.I.	
Intention to Treat Analysis	201/836, 24.1	166/835, 19.9	4.2 (0.2, 8.2)	0.34
Per Protocol Analysis	172/791, 21.7	140/801, 17.5	4.3 (0.3, 8.1)	0.36
SECONDARY OUTCOME: RELAPSE RATE				
Among those cured on day- 4	44/635, 6.9	41/669, 6.1	0.8 (−1.9, 3.5)	p = 0.6

Abbreviations:

m/n: numerator/denominator

CI: Confidence Interval

Among clinical failures on day- 4, 82.9% (160/193) in the placebo group and 83.9% (131/156) in the amoxycillin group had wheeze with or without any sign of hypoxia (oxygen saturation on pulse-oximetry <90%), severe pneumonia, very severe disease or axillary temperature >101°F. Only those with associated wheeze at the time of failure were at increased risk of having respiratory rate above age specific cut-off in the placebo group (OR = 6.0, 95% CI: 2.6–13.7; p value <0.001) as well as in the amoxycillin group (OR = 4.4, 95% CI: 1.7–11.2; p value = 0.001).

There was also no difference by group in the proportion of clinical relapses, among those diagnosed as cure on day- 4 (Table 2).

### Secondary laboratory Outcome

RSV antigen was positive in 5.6% (88/1558) at enrolment (Table 1).

### Risk factors associated with clinical failure

Significant univariate association of clinical failure were found with current history of fever and use of liquid petroleum gas for cooking at home (Table 3), but these were not significant in the multiple regressions. Variables which had significant association in both univariate and the multiple logistic regression model with clinical failure were current history of vomiting, past history of use of bronchodilator, excess respiratory rate of >10 breaths per minute above age specific cut-off and non-adherence (Table 4); the adjusted odds ratio for clinical failure with the use of placebo was 1.28 (95% CI: 1.01 to1.62; p value = 0.04).

**Table 3:** Association of clinical failure with different characteristics of patients (univariate Intention to Treat Analysis).

Variables	Clinical failure	Clinical Cure		Adjusted^2^	p-value
	(N = 367)	(N = 1304)		odds ratio, 95% C.I.	
	number, %	number, %			
Female	127, 34.6	473,36.3		0.9(0.7,1.2)	0.6
Age 2–11months	157,42.8	497,38.1		1.2(1.0,1.5)	0.11
Malnutrition*	9, 2.5	57, 4.4		0.6(0.3,1.1)	0.10
**History of Current Illness**					
Duration of illness > = 3days	293, 79.8	992,76.1		1.2(0.9,1.7)	0.14
Fever	230, 62.7	707, 54.2		1.4(1.1,1.8)	0.004
Vomiting	102, 27.8	244, 18.7		1.7(1.3,2.2)	<0.001
Cough	366,99.7	1297,99.5		2.0(0.2,16.1)	0.5
Diarrhea	3, 0.8	10, 0.8		1.0(0.3,3.8)	0.96
Difficult breathing	0	2, 0.2			0.5
**History of Allergic disorders**					
Asthma	45, 12.3	171, 13.		0.9(0.7, 1.3)	0.7
Allergic rhinitis	18, 4.9	91, 7.0		0.7(0.4, 1.2)	0.2
Eczema	2, 0.5	8, 0.6		0.9(0.2, 4.1)	0.9
Urticarial rash	0	11, 0.8			0.08
Use of Bronchodilator	107, 29.2	259, 19.9		1.7(1.3, 2.2)	<0.001
**History of exposure Pollutants**					
Smoker in family	127, 34.6	388, 29.8		1.3(1.0, 1.6)	0.06
Biomass fuel use for cooking	162, 44.1	534, 41.0		1.1(0.9, 1.4)	0.3
LPG use for cooking	211, 57.5	837, 64.2		0.8(0.6, 1.0)	0.02
**Examination**					
Respiratory Rate >10 units of cut-off	172, 46.9	500, 38.3		1.4(1.1, 1.8)	0.003
Wheeze (On Auscultation)	320, 87.2	1101, 84.4		1.3(0.9, 1.8)	0.2
RSV positive^1^	16/349, 4.6	72/1206, 6.0		0.7(0.4, 1.3)	0.3
**Non-Adherence** 1	34/346, 9.8	16/1296, 1.2		8.5(4.6,15.6)	<0.001

* Weight-for-height<−3SD

1 For these variables, the results are expressed in fractions as their denominators are different.

2 Adjusted for intervention effect.

*Abbreviations:*

LPG: Liquid Petroleum Gas

RSV: Respiratory Syncitial Virus

CI: Confidence Interval

**Table 4:** Multivariable logistic regression model for the factors associated with Clinical failure on Intention to Treat Analysis.

Variables	Crude Odds Ratio (95% C.I.)	Adjusted odds ratio (95% CI)	p value of adjusted odds' ratio
**Intervention with Placebo**	1.3 (1.03, 1.66)	1.28 (1.01, 1.62)	0.04
**History of Current Illness**			
Vomiting	1.7 (1.3,2.2)	1.50 (1.14, 1.98)	0.004
**History of Allergic disorders**			
Use of Bronchodilator	1.7(1.3, 2.2)	1.72 (1.31, 2.26)	<0.001
**Clinical Examination**			
Respiratory Rate>10 breaths per minute above cut-off	1.4 (1.1, 1.8)	1.51 (1.18, 1.92)	0.001
**Non-adherence**	9.0 (4.9, 16.4)	8.24 (4.46, 15.20)	<0.001

Abbreviation:

CI: Confidence Interval

### Adverse reactions

These had similar frequencies in both arms. There were no deaths and 30 hospitalisations (15 in each group). There were 11 cases of nausea and mild vomiting, 2 cases of diarrhoea with some dehydration, 9 cases of diarrhoea with no dehydration, 1 rash without itch, and 1 case of tremors.

## Discussion

This was a randomised, double blind placebo controlled trial to assess whether children of non-severe pneumonia with wheeze can be effectively managed without antibiotics. We have found that clinical failure was associated with placebo treatment (adjusted OR = 1.28, 95% CI: 1.01 to1.62). Other variables associated with clinical failure on multivariable analysis were history of vomiting, past history of use of bronchodilators, respiratory rate >10 breaths per minute above age specific cut off and non-adherence (Table 4). Therefore, there is no need for modification of the current WHO guidelines for the treatment of pneumonia [10].

This study was designed as an equivalence trial [9], [11] to get as close to the truth as possible with unbiased results. Our pre-specified value of 5% defined the smallest difference (excess) in clinical failure rates with the use of placebo that would be of clinical interest. In the ITT as well as per protocol analysis, the estimated difference in clinical failure was <5% but the confidence interval covered points that lie outside the equivalence range (upper confidence limit is greater than +δ). So the differences of potential clinical importance remain a real possibility and equivalence cannot be safely concluded on the basis of “confidence interval” approach. We used the other commonly used statistical approach of TOST to avoid any ambiguity in assessing the therapeutic equivalence. The TOST approach suggests that the hypothesis of in-equivalence cannot be disproved, which would then favour the use of antibiotics. The study was originally planned to have a power of 90%, but with the estimated overall failure rate of 22% and average sample of size of 835.5, the power of current study is 79.5%. It is recommended that a further study should be planned with a greater power using the estimates of failure rate of the current study.

Clinical failure as defined in this study was a composite measure. Since presence of hypoxia, severe pneumonia and very severe disease were responsible for similar proportions of clinical failures in both the treatment groups, and since >80% of clinically failed had wheeze along with respiratory rate above the age specific cut-off, failure was possibly due to inadequate bronchodilator therapy. However, theoretically clinical failure due to inadequate antimicrobial therapy cannot also be ruled out. We used amoxycillin that is affective against most strains of *Haemophilus influenzae* and *Streptococcus pneumoniae*, which are major causes of bacterial pneumonia [12]. Since clinical failures for reasons other than fast breathings were similar in the placebo as well as antibiotic groups, it is unlikely that these subjects would have benefited with antibiotics. On the contrary, they possibly require intensive bronchodilator therapy as recommended in the guidelines for management of cases of asthma without hypoxia [13].

Previous trials of antibiotics for treatment of non- severe pneumonia have documented respiratory rate >10 breath per minute above age specific cut off as a predictor of clinical failure [5], [6], as reported in the current study. A study from Pakistan reported temperature >100°F at baseline in wheezers with pneumonia as predictive of subsequent deterioration [14] as seen by us in univariate analysis. We have not found this relationship in multivariate analysis. Therefore, fever at baseline cannot be taken as an absolute criterion for giving antibiotics. In our study 4.9% of children with clinical failure had fever above 101°F, which could be either due to bacterial or viral etiology. Those with bacterial etiology of fever could be benefited with antibiotic therapy.

Wheezing is commonly associated with viral infections, particularly due to RSV [15]. However, the sample size of RSV positive cases in this study was too small to provide a precise estimate of the association in this particular sub-group. The proportion of RSV positivity was similar to a previous study conducted in same sites, and was low [5]. Lack of association between clinical failure and RSV could be due to the small number of RSV positive patients in the current study.

In our study past history of asthma and/or use of bronchodilators was not an exclusion criteria. This was done because in young children there are multiple causes of wheezing and asthma is just one of them. Thus, we have increased the generalizability of our results. We have found that, controlling for the use of antibiotic in logistic regression model, children with past history of use of bronchodilators are at increased risk of clinical failure, which is most likely due to inadequate bronchodilator therapy. In this sub-group of children further study is needed to define the benefit of concomitant antibiotic therapy when they get fast breathing, in the range of WHO-defined non-severe pneumonia.

We have found that among cases of non-severe pneumonia and wheeze, the respiratory came back below age specific cut-offs in 46% (1605/3487) children and thus there was no need to prescribe antibiotic. Similar findings have been reported from Pakistan [15]. The IMCI guideline also recommends use of bronchodilators among wheezers before deciding to treat them as cases of pneumonia with antibiotics. Implementation of IMCI guidelines in ambulatory care settings in India as well as other developing countries with result in a substantial reduction in prescription of antibiotics for non-severe pneumonia.

From this study we have found that treating 24 cases of non-severe pneumonia with wheeze with 3-days of oral amoxycillin along with bronchodilators will avert 1 clinical failure. With these NNT, in the developed countries treating non-severe pneumonia with wheeze without antibiotics may be considered since vaccination against *Haemophilus influenzae* and *Streptococcus pneumoniae*, are a part of their Expanded Program of Immunization (EPI). However, these vaccines are not a part of the EPI program in most developed countries, hence we have to continue to treat such children with antibiotics as well as bronchodilators to avert clinical failures as a result of bacterial pneumonia. For a 12.5 Kg child, oral amoxycillin will cost just 0.3 USD [16] and thus in 7.2 USD one failure can be averted. Given the incidence rate of 536 non-severe acute lower respiratory infections/1000 child years from India [17] of which about 13% will have also wheeze [5] this translates into an annual cost of USD 315,0000 on oral amoxycillin in India with about 1 billion population of which 15% are below 5 years of age. This cost cannot be averted for now as we found that treatment with placebo is not equivalent to that of 3-days of oral amoxycillin in such cases.

The strength of the study was that it was a multi-centric, double blind, placebo controlled trial, with good adherence and minimal follow-up in spite of ambulatory study design. Since we were using placebo to treat children of non-severe pneumonia, where antibiotics have been recommended by the WHO [10], we had to exclude those with radiological pneumonia from the trial to avoid exposing them to risky placebo treatment arm. While this has maximized the principle of avoiding harm to the patients from ethical as well as good clinical practises point of view, it has also reduced the generalizability of our findings. Since we have concluded that treatment with placebo and amoxycillin is not equivalent we have actually benefited children with radiological pneumonia by treating them with standard guidelines that does include antibiotics. Baseline and outcome variables were homogeneous across the sites. Since there was misinterpretation of radiological pneumonia, there were 3 cases of protocol deviations. We also did not investigate for microbial profile, as this is not done in routine community practise.

To conclude, treating children with non-severe pneumonia and wheeze with a placebo is not equivalent to treatment with oral amoxycillin since even though the estimated difference in clinical failure was <5% but the confidence interval covers points, which lie outside the equivalence range (upper confidence limit is greater than +5%). Perhaps more aggressive bronchodilator therapy will also benefit such patients, especially those with history of prior use of bronchodilators. Patients with respiratory rate >10 breaths per minute above cut-off should be studied further for the benefits of antibiotic therapy.

## Supporting Information

Checklist S1 CONSORT Checklist
(0.04 MB DOC)

Protocol S1 Trial Protocol
(0.58 MB DOC)
