# Supplementary material for: Does 3-Day Course of Oral Amoxycillin Benefit Children of Non-Severe Pneumonia with Wheeze: A Multicentric Randomised Controlled Trial
Source: PLoS One. 2008 Apr 23;3(4):e1991. doi: 10.1371/journal.pone.0001991 (PMC2292255; doi:10.1371/journal.pone.0001991)
Supplement: Protocol S1 — Trial Protocol (0.58 MB DOC) [file pone.0001991.s002.doc]

# Revised Protocol

**Date of revision: 15 January 2004**

# Randomized double blind placebo controlled trial of amoxycillin in the treatment of non-severe pneumonia with wheeze in children aged 2- 59 months of age: A multi-centric Study

IndiaClen - INCLEN- Childnet collaborative Multicentre project

Indiaclen short course amoxycillin therapy for pneumonia with wheeze - II

**(ISCAP- II)**

IPHIDE RESEARCH INITIATIVE

Date ................................ Protocol # ......................

**INCLEN Inc.**

**IRB PROTOCOL "FACE SHEET"**

**Project Title:** Randomized double blind placebo controlled trial of amoxycillin in the treatment of non-severe pneumonia with wheeze in children aged 2-59 months of age

**Funding Agency or Sponsor:** Indiaclen Grant #..............

---------------------------------------

**Address:** 3600 Market Street, Philadelphia PA 19104-2644, USA

**Principal Investigator, Title & Department:** Dr. Shally Awasthi,

Professor,

Department of Pediatrics,

**Mailing Address:**  Department of Pediatrics,

C.S.M. Medical University (Upgraded K.G.M.C.),

Lucknow- 226003

**Telephone:** 91-0522-2257329

**Fax:** 91-0522-2257674

**E-Mail:** sawasthi@sancharnet.in

**Other Investigators:**

# Site Clinical

Delhi Dr. S.K Kabra

Chandigarh Dr. Sunit Singhi

Nagpur Dr. A.K Niswade

Trivandrum Dr. R.M Pillai

Vellore Dr. Raghupathy

Mumbai Dr. M. Kulkarni

Lucknow Dr. S Awasthi

Chennai Dr. Luire Ravi chellah &sardha suresh

Bhopal Dr. R Diwedi

**PLEASE ANSWER THE FOLLOWING QUESTION**

1 ................ YES ......… √....... NO This project is to be undertaken as part is a previously approved Program grant. Grant Number, Project Title and Director.

2 ......... √....... YES ................ NO Does the project involve the administration of personality tests. Inventories, or questionnaires? IF YES, provide the name of the standard tests or questionnaire or 3 copies of the proposed tests.

3 ......... √....... YES ................ NO Does the project involve the use or drawing of human blood, blood products, tissues or body fluids?

4 ................ YES ......... √....... NO Does the project involve administration of ionizing radiation to subjects for other than clinical purposes?

5. ................ YES ........ √...... NO Does this project involve the testing of investigational drugs or devices? IF YES, provide: Name of drug or Device: ................. Name of Manufacturer: ....................... if this protocol involves the administration of medications to humans for research purposes (not part of general clinical practice), you must obtain an authorization.

6. Human Subjects would be involved in the proposed activity either:............. None of the following, or including .......√....... minors, ...............fetuses, ................. abort uses, ...........Pregnant women, ..............Prisoners, ............... mentally retarded, ............... mentally disabled, ................. HIV ................ Positive subjects

Signature: Principal Investigator: Shally Awasthi

Chairperson

The signature of each department chairperson with faculty involved is required.

A Dean's signature must be obtained if the investigator is also the chairperson.

**Index**

**List of Contents Pages**

1. Title Page 1

2. List of participating sites 6-7

2.1 Executive Summary 8-16

3. Introduction 17-21

Review of Literature 17-19

Intended use of study findings 19

Study design and location 20

Objectives 20-21

Outcome 21

Approach 21

4. Procedures and Methods 22-25

Design 22

Stake holders 22-23

Cost-effectiveness analysis 23-24

Time line 25

5. Population 25-33

Description of site 25

Case definitions 25-28

Inclusion criteria 28

Exclusion criteria 28-29

Sample size and sampling technique 29-30

Randomization 30-31

Enrollment 32-33

Consent 33

6. Variables and interventions 33-69

Variables 34

Outcome assessment 34-36

Study treatment 36-37

Change of antibiotic therapy 38

Non-compliance 38

General Supportive Care 39

Discontinuation of study 39-40

Study instruments 40-68

Quality Control 69

Training of study personnel 69

7. Data Analysis and management 69-72 Intermediate reviews and analysis 70-71

Bias 71

Study management at the study site 71-72

Project monitoring and reviews 72

Limitations of the study 72

8. Handling of unexpected or adverse events 73-78

9. Committee 75-76

10. Dissemination, notification and reporting 77-78

11. Ethical 78

12 Bibliography 78-79

13. Appendix 80-85

# Randomized double blind placebo controlled trial of amoxycillin in the treatment of non-severe pneumonia with wheeze in children aged 2- 59 months of age:

# A multi-centric double–blind trial

IndiaClen - INCLEN- Childnet collaborative Multicentre project

**Project coordinator:**

Dr. Shally Awasthi,

INCLEN Childnet co-ordinator

Professor, Dept. of Pediatrics & Institute of Clinical Epidemiology

Upgraded KG Medical College, Lucknow (UP), India -226003

**Members Central Co-ordination Team:**

Dr. Girdhar Awarwal, Biostatistician, Lucknow, India

Mr. Jaideep Singh, Computer Programmer, Lucknow, India

Mr. Hasibur Rehman, Secretarial Assistance

**Technical advisers**:

Dr. Shamim Qazi - WHO, Geneva

Prof. Stephen Walter - McMaster, Canada

**Funding agencies:**

USAID - Indian Mission

Indiaclen Infectious disease initiative

**Data Monitoring Committee:**

Dr. JN Pande, AIIMS, New Delhi - Chair person

Dr. Indu Waklhu, Professor and Ex-HOD, Pediatrics, KGMC, Lucknow

Dr. NC Saxena, Addl DG, RCH, ICMR, New Delhi

Dr Stephen Walter

# Officer In Charge (Randomization) Dr. Sanjay Khattri, Dept of Pharmacology, Upgraded KGMC, Lucknow

**Biostatistician**  Dr. GG Agarwal

Undertaking by Investigators

*I, ____________________________________(write name in blocks), agree with the protocol and I have read and understood it. I give in writing that I will work according to the protocol.*

*.S.No Name Signature Date*

1. Dr.S.K.Kabra (New Delhi)
2. Dr.SunitSinghi (Chandigarh)
3. Dr.A.K.Niswade (Nagpur)

4 Dr.R.M.Pillai (Trvandrum)

1. Dr.Madhuri Kulkarni (Mumbai)
2. Dr.Srinivas (Chennai)
3. Dr.RashmiDiwedi (Bhopal)
4. Dr.Raghupathy (Vellore)

9 Dr.ShallyAwasthi (Lucknow)

Randomized double blind placebo controlled trial of amoxycillin in the treatment of non-severe pneumonia with wheeze in children aged 2- 59 months of age: A multi-centric double–blind trial

## List of Investigators and Study Sites

| City Hospital Investigator (Clinical) |
| --- |
| 1. New Delhi AIIMS Dr. Sushil Kabra |
| 2. Nagpur Govt. Medical College Dr. Niswade |
| 3. Vellore CMC Dr. Raghupathy |
| 4. Trivandrum Medical College Dr. RajMohan Pillai |
| 5. Bombay Sion (BMC) Prof. Madhuri Kulkarni  Dr. Vaishali |
| 6. Bhopal GMC Dr. Rashmi Diwedi |
| 7 Chennai MGR University Dr. Sriniwasan |
| 8. Chandigarh PGI Prof. Sunit Singhi |
| 9. Lucknow KGMC Dr. Shally Awasthi |

**2.1 Executive Summary**

**Backgroud and Literature review**

The World Health Organizations acute lower respiratory infections (ALRI) management algorithms depend primarily on two key clinical signs: elevated respiratory rate and chest indrawing. The current Integrated management of childhood infections (IMCI) algorithm prescribes that children with wheeze and fast breathing presenting to first level health facilities are given antibiotics if they continue to have fast breathing after two doses of bronchodilator. The primary purpose of the algorithm is to prevent mortality due to bacterial pneumonia. However, an unknown proportion of children managed in this fashion will have a viral related wheezing illness or asthma rather than pneumonia. Although it is unlikely that wheezing syndromes are a significant cause of mortality for children in developing countries, these algorithms are likely to result in unnecessary administration of antibiotics as well as inadequate treatment of recurrent wheezing illness (1).

In both developed and developing countries respiratory syncytial virus is the predominant etiological agent responsible for bronchiolitis and wheezing illness in the first two years of life(2,3). Moreover data from several studies demonstrates that respiratory syncitial virus (RSV) infection and the bronchiolitis syndrome are a major component of the total ALRI in children living in developing countries(2). The relevant literature on therapy includes studies in which children were labelled as bronchiolitis and others in which children were classified as wheezing illness.

We do not have clear evidence about whether antibiotics can be withheld in some categories of children with wheeze. It is clear that wheeze can occur in bacterial infection and in addition co-infection with virus and bacteria has been well demonstrated in several studies of pneumonia etiology in children. Although some studies have found that children with more severe disease or who are blood culture positive are more likely to be febrile at presentation, this sign is not sufficiently sensitive or specific to determine whether antibiotics should be administered (4,5).

There has been extensive debate about whether infants and young children in the first year of life respond to bronchodilator therapy. Proposed reasons for a lack of response have included: immaturity of bronchiolar smooth muscle, increased dynamic airway closure and relatively larger degrees of mucosal edema. A literature search between 1980 and 2000 reveals five randomized placebo controlled trials of beta agonist administered to acutely wheezing infants in which clinical outcomes were determined (6-10). Overall these studies support the hypothesis that children aged less than 12 to 18 months are less responsive to bronchodilator therapy than older children. However, they also demonstrate that use of inhaled short acting bronchodilators for the acute treatment of wheeze offers some benefits for clinical outcomes even in this young age group. The benefits of beta agonists may be restricted to children with recurrent wheezing and at most provide a very small clinical benefit.

Wet nebulizers are currently widely used for the delivery of bronchodilator therapy to young children in emergency and outpatient settings. However, the use of metered dose inhalers (MDIs) with holding chambers for this purpose has a number of strategic advantages, especially in the primary care setting. The nebulizer is a costly piece of equipment and requires a power source as well as maintenance to perform as it was designed. A holding chamber and MDI is a less sophisticated piece of equipment that is cheaper to purchase and requires no power source.

A recent systematic review has compared the effectiveness of holding chamber devices and wet nebulizers in the acute management of asthma(11). A total of 16 trials were identified including 7 that were conducted in children, mainly aged over 3 or 4 years. Trials that reported data from children demons-trated that, compared with wet nebulizers, administration of beta agonist aerosols by MDI with holding chamber resulted in less time spent in the emergency room, better oxygenation and a lower pulse rate. The two delivery methods did not differ in their effects on the rate of improvement in lung function or the need for admission to hospital. The authors of this meta-analysis concluded that for treatment of severe bronchoconstriction in children, four actuations of the MDI into the holding chamber given each 10-15 minutes until recovery, was a safe and effective dosage regimen.

Bronchodilator administered by a holding chamber with an attached mask has recently been shown to be equivalent to bronchodilator administered by wet nebulizers in terms of its effect on clinical asthma score, respiratory rate, pulse and oxygen saturation in 42 young children (aged 10 months to 4 years) with severe wheezing(12).

The efficiency of plastic holding chambers for delivery of aerosol is seriously impaired by the electrostatic charge that exists within these devices (13-14). Aerosol deposi-tion is improved by priming the devices with active drug or placebo or by soaking the device in an ionic detergent prior to use. The holding chambers that have been studied in most clinical trials are all proprietary devices. However some investigators have assessed the value of more readily available, non-proprietary devices. In 88 South African children presenting with acute asthma, a sealed 500 ml plastic bottle was as effective for delivery of fenoterol hydrobromide as a commercial spacer(15). Equivalence was demonstrated for clinical score and lung function improvement. Unsealed plastic bottle and a polystyrene cup were less effective than the sealed bottle or the commercial spacer. In summary, holding chambers are at least as effective as wet nebulizers for the adminis-tration of bronchodilators to children of all ages. For young children, a mask is necessary to connect the chamber to the child’s face.

*In the current study we will use amoxycillin for three days in the conventional dose of thrice a day. Literature worldwide shows equal efficacy of shorter courses of antibiotics.*

*Evidence in favour of shorter courses of antibiotics:*

1. *An observational study conducted in children between 2 to 59 months with non-severe pneumonia at King George’s Medical College, Lucknow from 8.8.97 to 23.10.97, showed clinical cure in 77.65% cases with six doses of oral amoxycillin (16)*
2. *An intervention randomized trial in Santo- Domnigo, Dominican Republic was conducted in children between 6 to 59 months from Oct.1999 to July 2000. Two treatment arms were designed to receive short course, high dose or long duration, normal course of amoxycillin. The results showed increased compliance and decreased bacterial resistance in the short course group (17).*
3. *A multicentric randomized trial was conducted in Pakistan from Oct. 1999 to April 2001 to assess the efficacy of three days versus five days course of amoxycillin in children between 2 to 59 months with non-severe pneumonia. The results showed equal response and failure to treatment in children in both the arms (18).*

*4. A study from Bangladesh has documented under-dosing in children suffering from moderate ALRI (non-severe pneumonia). Among 358 episodes analyzed, more than half them resulted in receiving 6 instead of 10 doses of co-trimoxazole without any change in the cure rates. There was just 1 treatment failure. It was concluded that shorter courses of antibiotics might be effective for treatment of non-severe pneumonia (19).*

Application of these data will allow improvement in the current algorithms for the management of ALRI with wheeze. The benefits of such improvements will primarily be in better acute management of children with wheeze. The question of which children with wheeze can safely have antibiotics with-held remains a major question for further .

The present study plans to evaluate the role of antibiotic in children with non-severe pneumonia presenting with wheeze. It will define the patient and disease characteristics associated with clinical failure and the need for antibiotics. The results of the study will formulate policy to use antibiotics in children with non-severe pneumonia and wheeze.

**Reference**

1. Sachdev HPS, Mahajan SC, Garg A. Improving antibiotic and bronchodilator rescription in children presenting with difficult breathing: experience from an urban hospital in India. Indian Pediatr 2001; 38: 827-838.
2. Selwyn BJ. The epidemiology of acute respiratory tract infection in young children: Comparison of findings from several develop-ing countries. Coordinated Data Group of BOSTID Researchers. Rev Inf Dis 1990; 12: S870-S888.
3. Glezen P, Denny FW. Epidemiology of acute lower respiratory disease in children. N Engl J Med 1973; 288: 498-505.
4. El Radhi AS, Barry W, Patel S. Association of fever and severe clinical course in bronchio-litis. Arch Dis Child 1999; 81: 231-234.
5. Weber MW, Dackour R, Usen S, Schneider G, Adegbola RA, Cane P, et al. The clinical spec-trum of respiratory syncytial virus disease in the Gambia. Pediatr Infect Dis 1998; 17: 224-230.
6. Prahl P, Peterson NT, Homsieth A. Beta 2-agonists for the treatment of wheezy bron-chitis. Ann Allergy 1986; 57: 439-441.
7. Alari AJ, Lewander WJ, Dennehy P, Seifer R, Mansell AL. The efficacy of nebulized metaproterenol in wheezing infants and young children. Am J Dis Child 1992; 146: 412-418.
8. Bentur L, Canny GJ, Shields MD, Karem E, Schuh S, Reisman JJ, et al. Controlled trial of nebulized albuterol in children younger than 2 years of age with acute asthma. Pediatrics 1992; 89: 133-137.
9. Mallol J, Barrueto L, Girardi G, Munoz R, Puppo H, Ulloa V, et al. Use of nebulized bronchodilators in infants under 1 year of age: Analysis of four forms of therapy. Pediatr Pulmonol 1987; 3: 298-303.
10. Chavasse RJ, Bastian-Lee Y, Richter H, Hilliard T, Seddon P. Inhaled salbutamol for wheezy infants: A randomized controlled trial. Arch Dis Child 2000; 82: 370-375.
11. Cates C J, Rowe BH, Bara A. Holding chambers versus nebulisers for beta-agonist treatment of acute asthma. The Cochrane Library, 2: 2002.
12. Mandelberg A, Tsehod S, Houri S, Gilad E, Morag B, Priel IE. Is nebulized aerosol treatment necessary in the pediatric emer-gency department? Chest 2000; 117: 1309-1313.
13. Kenyon C, Thorsson L, Borgstrom L, Newman S. The effects of static charge in spacer devices on glucocorticosteroid aerosol deposition in the asthmatic patient. Eur Respir J 1998; 11: 606-610.
14. Wildhaber JH, Devadason SG, Eber E, Hayden MJ, Everard ML, Summers QA, et al. Effect of electrostatic change, flow, delay and multiple actuations on the in vitro delivery of salbutamol from different small volume spacers for infants. Thorax 1996; 51: 985- 998.
15. Zar H, Brown G, Donson H, Braithwaite N, Mann M, Weinberg E. Homemade spacers for bronchodilator therapy in children with acute asthma: A randomized trial. Lancet 1999; 54: 979-982.
16. Awasthi S. Clinical response to two days oral amoxycillin in children with non-severe pneumonia. Indian Pediatrics 2000;37:301-306
17. Schrag S, Pena C, Fernandez J, Sanchez J, Gomez V, Peraz E, Feris J, Besser R. Effect of short course, high dose of amoxycillin therapy of resistant pneumococcal carriage. JAMA 2001;286:49-56
18. Clinical efficacy of three days versus five days of oral amoxycillin for the treatment of childhood pneumonia: a multicentric double- blind trial. Lancet July 2002: 1-7
19. Francisco AD, Chakraborty J. Adherence to co-trimoxazole treatment for the acute lower respiratory tract infections in rural Bangladeshi children. Annals of Tropical Pediatrics 1998;17-21
20. GCP Guidelines

###### Objectives and Hypothesis

For the randomized placebo controlled trial, the primary hypothesis is that the use of oral amoxycillin for three days would be as effective, in terms of clinical cure on day 4 as compared to use of oral placebo.

## Primary

To compare the proportion of children aged 2 to 59 months presenting with non-severe pneumonia with wheezing whose respiratory rate does not fall below the age specific cut-off after three doses of nebulized salbutamol, that achieve clinical cure on day 4 on 3 day of treatment with oral amoxycillin versus placebo

## Secondary

Among all cases of non-severe pneumonia with wheeze

1. To assess the proportion of children aged 2 to 59 months presenting with non-severe pneumonia with wheezing audible wheeze

2. To assess the proportion of children aged 2 to 59 months presenting with non-severe pneumonia with wheezing who respond to up to three doses of nebulization with salbutamol**.**

Among cases of non-severe pneumonia with wheeze aged 2 to 59 months who respond to three doses of salbutamol

3. To assess the proportion of who fail therapy at day 4 of the initial successful bronchodilator therapy with inhaled salbutamol

4. To assess the proportion relapse at day 11- 14 of the initial successful bronchodilator therapy with inhaled salbutamol

5.To compare the cost of treatment of clinical failures and relapses among those treated with oral salbutamol.

6 .To assess the association of bronchodilator response with age, season, number of previous wheezing episodes, audible versus auscultatory wheeze and family history of asthma.

7.To assess the association of relapse in children who showed improvement after being treated with inhaled salbutamol, with age, respiratory syncytial virus (RSV) isolation, season, number of previous wheezing episodes, audible versus auscultatory wheeze and family history of asthma.

Among cases of non-severe pneumonia with wheeze aged 2 to 59 months who do not respond to three doses of salbutamol

8.To compare the proportion of children who are judged to be clinically cured after 3 days of treatment but who relapse within the next 11-14 days of observation on 3 day treatment with oral amoxycillin versus placebo.

9.To compare the cost of treatment of clinical failures and relapses among those treated with oral amoxycillin or placebo.

10. To assess the association of clinical failure on day 4 with age, respiratory syncytial virus (RSV) isolation, season, number of previous wheezing episodes, audible versus auscultatory wheeze, family history of asthma and randomization to amoxycillin therapy.

**Study Design and methods**

***Setting***

The study will be conducted from the outpatient’s department or emergency rooms in large secondary as well as tertiary care hospitals in India in 9 cities of Bhopal, Chandigarh, Chennai, Lucknow, Mumbai, Nagpur, New Delhi, Trivandrum and Vellore.

*Design*

This will be a multicentric, randomized, double blind efficacy trial. Block randomization will be done in Dept. of Pharmacology, Upgraded KGMC, Lucknow, which is not the coordinating center for the trial. Blocks will be generated in mixed batches. Dr. Sanjay Khattri, who is not the biostatistician responsible for the design and analysis of the study, nor is he on the data monitoring committee will be responsible for random allocation (Randomization Officer-in-Charge). The medicines will be then placed in serially numbered envelopes by the pharmacy according to the intervention type determined by the pharmacy. Two hundred and twenty five random numbers will be generated in blocks of varying lengths for each of the nine sites.

Medicines for three days-placed in colored envelopes will contain either amoxycillin or amoxycillin placebo. The drug will be given three times a day. These envelopes will be serially labeled for each site and mailed to the coordinating center, Lucknow. These pre-prepared sealed envelopes containing the treatment assignment for the entire period of the study will be sent to the sites at the beginning of the study.

Children who come to the OPD with cough, and or fast breathing will be screened. Those who meet the inclusion criteria and have no exclusion criteria will be invited to participate in the study. Patients with wheeze will be given three doses of salbutamol inhalation by MDI (metered dose inhalation). Those who improve and their respiratory rate falls below the age specific cut-off will be sent home on oral bronchodilator therapy. Children still having fast breathing after three doses of bronchodilator will be subjected to X-ray film of chest for diagnosis of pneumonia. Pneumonia will be diagnosed if there are paranchymal infilterates or patch of consolidation. Children with radiological pneumonia will be treated according to the standard hospital treatment guidelines. Those without radiological pneumonia will be invited to participate in the study and randomized to receive antibiotics or placebo along with oral salbutamol in three divided doses.

The patients presenting in the out atient department be followed up on day 4, and 11-14 for reassessment. A child will be labelled as failed if he continues to have rapid respiration and or wheezing. S/He will be treated according to practice of the hospital.

At enrollment a baseline assessment will be done after the patient’s guardian consents for the study. A nasopharyngeal aspirate will be taken for RSV detection. There will be two mandatory follow-up visits, on day 4, and day 11-14 after randomization. Those who fail to report to the health facility on the appointed day will be visited at home. During hospitalization and each follow-up visit, the child will be assessed for the following**:**

- Clinical status including respiratory rate, heart rate, temperature and presence or absence of wheeze.
- Primary and secondary outcomes.
- Presence of any adverse events.

During the course of the study any patient who fails the therapy will be treated as per the treatment protocol of the hospital and kept under follow-up as per the study protocol.

Outcomes

*Primary*

- ***Clinical Cure***: Respiratory rate below ages specific cut-off (<50 bpm in infants <1 year and <40 bpm in ages 12 – 59 months) and absence of auscultatory as well as audible wheeze.

*Secondary*

*Response to nebulization*: Respiratory rate below age specific cut-off (<50 bpm in infants <1 year and <40 bpm in ages 12 – 59 months) after a maximum of three doses of nebuliztion with salbutamol.

Auscultarory wheeze may or may not be present. But there is no audible wheeze.

- ***Clinical failure of therapy:*** Clinical deterioration as indicated by the occurrence of one or more of the following:

1. Any signs of severe pneumonia or severe disease: chest in drawing, convulsions, drowsiness or inability to drink at any time.
2. Respiratory rate above age specific cut-off on day 4 or after that (with or without wheeze).

3. Oxygen saturation on pulse oximetry <90% on day 4 or after that.

4. Documented axillary temperature > 101 degrees Fahrenheit

In addition, children who die within the follow-up period of 14 days or are lost to follow-up on day 4 will also be considered as failed.

***Clinical relapse at day 7- 15***: Signs of severe pneumonia or very severe disease among cases who were clinically cures on day 4 follow-up.

**Inclusion and Exclusion criteria**

Included will be children aged 2-59 months seeking care spontaneously (not referred) at the outpatient units of the selected sites with complaints of cough, wheeze and or difficult breathing of less than 30 days of duration with respiratory rate above age-specific cut off point >= 50 bpm for children 2-11 months or >= 40 bpm in children 12- 59 months of age.

Excluded will be children with presence of danger sign or evidence of severe illness, received any documented antibiotic treatment in the past 48 hours, diagnosed asthmatic on maintenance therapy, complicating acute non-pulmonary or chronic illness, known drug allergy, hospitalization in the past 2 weeks, measles or history of measles within the last month, known immunodeficiency disorder, prior enrollment in the study, residing in areas not accessible for follow-up or whose guardian refuses to consent for the study and those with radiological Pneumonia on X-Ray.

**Sample size**

Two modes of therapies will be assumed to be equal if the failure rate in the new regimen is not more than 17% (difference is within 5%). So each site will be required to recruit and follow-up 225 cases in each arm in 18 months period. This is the minimum required sample size.

Data management and analysis

## The data from all the sites will be managed at the co-ordinating center. It will coordinate all project activities plus ensure quality of data. The primary data will be entered at the respective site and then secondary entry will be made at the co-ordinating site. The cleaned data will be analyzed at the co-ordinating center using standard software packages.

## For monitoring treatment failures and adverse reactions, a data monitoring committee DMC will be constituted that will comprise of members as renowned individuals with varied experiences, specifically in the areas of randomized trials and epidemiological study design and child health and acute respiratory infections and will be neither associated with study design, implementation, data collection, funding or donations for the work.

Audience and stakeholder participation

The primary audience for the work is the health care providers in India as well as the community. The latter is also the primary stakeholders. Since the trial is being done on children, the views of the parents or guardians will be taken. The subjects will be enrolled only after obtaining informed consent. They have full rights to withdraw the child from the study or go to an alternative health care provider anytime during the trial or thereafter. Besides assessing clinical cure and failures by objective criteria, the parents will specifically be asked if they feel that their child has improved or not. Parental assessment may differ from objective clinical assessment but is important as it has a direct bearing with continuation of therapy and follow-up in the real life situation.

# Dissemination

All data derived from this multicentre study will be the property of the investigators and the sponsors. The study will be subject of a medical research report compiled by the investigators. The sponsors via the steering committee may disclose the data derived from the study to any of their subsidiary firms, to other investigators and national or foreign drug regulatory authorities.

The investigators have the ultimate right and responsibility to publish. They are free to use the data derived from the study site for scientific purposes, but they should discuss any publications with the sponsors prior to release. The study sponsors recognize the right of the investigators to publish the results upon the completion of the study. The sponsors via the steering committee request that the investigators send a draft manuscript for publication to them for critical review prior to submission for the final version for publication. These draft manuscripts will be promptly reviewed within 1 month of submission. In case of difference of opinion between the sponsors and the investigator discussions will be held to be attempt to find a solution that satisfies both.

**Ethical Clearance**

# The local institutional ethics committee will clear the study first. Thereafter it will be submitted to the IndiaClen-USAID IRB for clearance since the US-India Mission (Infectious disease initiative) money will fund the work.

**TIME-LINE**

1. Finalize protocol, submit to RSC, IRB and secure funds March. – April 2003

2. Pre-trial preparations:

Organize purchase of drugs, and randomization, printing of instruments, preparing programs for data entry and management March – May 2003

3.Constitution and 1^st^ meeting of the Data monitoring committee May 2003

4. Quality assurance workshop May 2003

(For standardized training of appointed ROs at all sites in outcome assessment and data collection in Lucknow: - sending back project equipment and data collection forms)

5. Pilot (6-12 cases) Late May 2003

6. Recruitment in main study June 2003

7. Quality assurance site visits: June – July 2003

8. Interim analysis November 2003

9. 2^nd^ meeting of the data monitoring committee December 2003

10. Data cleaning and analysis Sept-

11. 3^rd^ meeting of the data monitoring committee and un-coding of the project December 2004

12. Report and manuscript writing workshop January 2005

The data monitoring committee can meet at any time on the call of the chairperson.

**3. Introduction**

**3.1.Literature review**

The World Health Organizations acute lower respiratory infections (ALRI) management algorithms depend primarily on two key clinical signs: elevated respiratory rate and chest indrawing. The current Integrated management of childhood infections (IMCI) algorithm prescribes that children with wheeze and fast breathing presenting to first level health facilities are given antibiotics if they continue to have fast breathing after two doses of bronchodilator. The primary purpose of the algorithm is to prevent mortality due to bacterial pneumonia. However, an unknown proportion of children managed in this fashion will have a viral related wheezing illness or asthma rather than pneumonia. Although it is unlikely that wheezing syndromes are a significant cause of mortality for children in developing countries, these algorithms are likely to result in unnecessary administration of antibiotics as well as inadequate treatment of recurrent wheezing illness (1).

In both developed and developing countries respiratory syncytial virus is the predominant etiological agent responsible for bronchiolitis and wheezing illness in the first two years of life(2,3). Moreover data from several studies demonstrates that respiratory syncitial virus (RSV) infection and the bronchiolitis syndrome are a major component of the total ALRI in children living in developing countries(2). The relevant literature on therapy includes studies in which children were labelled as bronchiolitis and others in which children were classified as wheezing illness.

**Acute Management of Young Children Presenting with Wheezing Illness**

We do not have clear evidence about whether antibiotics can be withheld in some categories of children with wheeze. It is clear that wheeze can occur in bacterial infection and in addition co-infection with virus and bacteria has been well demonstrated in several studies of pneumonia etiology in children. Although some studies have found that children with more severe disease or who are blood culture positive are more likely to be febrile at presentation, this sign is not sufficiently sensitive or specific to determine whether antibiotics should be administered (4,5).

There has been extensive debate about whether infants and young children in the first year of life respond to bronchodilator therapy. Proposed reasons for a lack of response have included: immaturity of bronchiolar smooth muscle, increased dynamic airway closure and relatively larger degrees of mucosal edema. A literature search between 1980 and 2000 reveals five randomized placebo controlled trials of beta agonist administered to acutely wheezing infants in which clinical outcomes were determined(6-10). Overall these studies support the hypothesis that children aged less than 12 to 18 months are less responsive to bronchodilator therapy than older children. However, they also demonstrate that use of inhaled short acting bronchodilators for the acute treatment of wheeze offers some benefits for clinical outcomes even in this young age group. The benefits of beta agonists may be restricted to children with recurrent wheezing and at most provide a very small clinical benefit.

**Delivery of Inhaled Bronchodilators**

Wet nebulizers are currently widely used for the delivery of bronchodilator therapy to young children in emergency and outpatient settings. However, the use of metered dose inhalers (MDIs) with holding chambers for this purpose has a number of strategic advantages, especially in the primary care setting. The nebulizer is a costly piece of equipment and requires a power source as well as maintenance to perform as it was designed. A holding chamber and MDI is a less sophisticated piece of equipment that is cheaper to purchase and requires no power source.

A recent systematic review has compared the effectiveness of holding chamber devices and wet nebulizers in the acute management of asthma (11). A total of 16 trials were identified including 7 that were conducted in children, mainly aged over 3 or 4 years. Trials that reported data from children demons-trated that, compared with wet nebulizers, administration of beta agonist aerosols by MDI with holding chamber resulted in less time spent in the emergency room, better oxygenation and a lower pulse rate. The two delivery methods did not differ in their effects on the rate of improvement in lung function or the need for admission to hospital. The authors of this meta-analysis concluded that for treatment of severe bronchoconstriction in children, four actuations of the MDI into the holding chamber given each 10-15 minutes until recovery, was a safe and effective dosage regimen.

Bronchodilator administered by a holding chamber with an attached mask has recently been shown to be equivalent to bronchodilator administered by wet nebulizers in terms of its effect on clinical asthma score, respiratory rate, pulse and oxygen saturation in 42 young children (aged 10 months to 4 years) with severe wheezing (12).

The efficiency of plastic holding chambers for delivery of aerosol is seriously impaired by the electrostatic charge that exists within these devices (13-14). Aerosol deposi-tion is improved by priming the devices with active drug or placebo or by soaking the device in an ionic detergent prior to use. The holding chambers that have been studied in most clinical trials are all proprietary devices. However some investigators have assessed the value of more readily available, non-proprietary devices. In 88 South African children presenting with acute asthma, a sealed 500 ml plastic bottle was as effective for delivery of fenoterol hydrobromide as a commercial spacer (15). Equivalence was demonstrated for clinical score and lung function improvement. Unsealed plastic bottle and a polystyrene cup were less effective than the sealed bottle or the commercial spacer. In summary, holding chambers are at least as effective as wet nebulizers for the adminis-tration of bronchodilators to children of all ages. For young children, a mask is necessary to connect the chamber to the child’s face.

In the present study, we will use the antibiotic for a short duration. We will administer amoxycillin for three days in the conventional dose of thrice a day. There is a lot of literature gathering worldwide about the efficacy of shorter courses of antiobiotics.

*Evidence in favor of short course of antibiotics (16-19)*

1. *An observational study conducted in children between 2 to 59 months with non-severe*

*pneumonia at King George’s Medical College, Lucknow from 8.8.97 to 23.10.97,*

*showed clinical cure in 77.65% cases with six doses of oral amoxycillin (16)*

1. *An intervention randomized trial in Santo- Domnigo, Dominican Republic was*

*conducted in children between 6 to 59 months from Oct.1999 to July 2000. Two*

*treatment arms were designed to receive short course high dose or long duration conventional course of amoxycillin. The results showed increased compliance and decreased bacterial resistance with shorter course (17).*

1. *A multi-centric randomized trial was conducted in Pakistan from Oct. 1999 to April 2001 to assess the efficacy of three days versus five days course of amoxycillin in children between 2 to 59 months with non-severe pneumonia. The results showed equal response and failure to treatment in children in both the arms (18).*

*4. A study from Bangladesh has documented under-dosing in children suffering from moderate ALRI (non-severe pneumonia). Among 358 episodes analyzed, more than half them resulted in receiving 6 instead of 10 doses of co-trimoxazole without any change in the cure rates. There was just 1 treatment failure. It was concluded that shorter courses of antibiotics might be effective for treatment of non-severe pneumonia (19).*

*The short course of antibiotic treatment has been successful in otitis media, sinusitis and tonsillopharyngitis in children.*

*Short courses have substantial benefits; it means lower cost of therapy, resulting in reduced expenditure on drugs in developing countries. Fewer doses would improve adherence, and would result in better tolerance. Also short course has been proposed as an alternative to reduce spread of resistance to pneumonia in cases in which antibiotic treatment is necessary. Short course reduces patient exposure to antibiotic selective pressure and may reduce antibiotic use in community, which often correlate with incidence of resistance. Studies in developing and developed countries have shown low compliance with antibiotics use. Using the drug for a shorter period is likely to increase the compliance with the prescribed regimen (24-26).*

*See appendix II for ISCAPI blinded results showing equivalence of two treatment types (one amoxicillin for three days and other amoxicillin for five days)*

Application of these data will allow improvement in the current algorithms for the management of ALRI with wheeze. The benefits of such improvements will primarily be in better acute management of children with wheeze. The question of which children with wheeze can safely have antibiotics with-held remains a major question for further research.

**3.2 Policy Relevance**

Unpublished data from the ongoing ISCAP study has shown that about 20% children WHO criteria of non-severe pneumonia have wheeze on auscultation. In one-half of them there is a past history suggestive of asthma, and hence they were excluded from ISCAP trial. The remaining half had broncho-spasm either as the first attack of bronchial asthma or bronchiolitis or bronchopneumonia. In all of these conditions, with the possible exception of bronchopneumonia, there is no role of antibiotics. In absence of clear guidelines to for or against the use of antibiotics among cases of non-severe pneumonia with wheeze, the practitioners often prescribe antibiotics in hospital settings. This results in over and possibly irrational use of antibiotics.

The present study plans to evaluate the role of antibiotic in children with non-severe pneumonia presenting with wheeze. It will define the patient and disease characteristics associated with clinical failure and the need for antibiotics. The results of the study will formulate policy to use antibiotics in children with non-severe pneumonia and wheeze.

**3.3 Study design and location**

Children presenting with non-severe pneumonia with wheeze will be nebulized at least thrice with salbutamol. Those whose respiratory rate falls below the age specific cut-off will be the part of an observational follow-up study. They will be sent home on oral salbutamol and followed up on days 4, and 11-14. Chest X-Ray will be done on children whose respiratory rate remains above the age specific cut-off after initial nebulizations. Those whose X-Ray does not show signs of paranchymal infiltrates or consolidation will be potential candidates for enrolment in the double blind placebo controlled trial.

The study will be conducted in nine medical colleges situated in New Delhi, Chandigarh, Lucknow, Mumbai, Bhopal, Nagpur, Chennai, Vellore and Trivandrum.

**3.4. Hypothesis**

For the randomized placebo controlled trial, the primary hypothesis is that the use of oral amoxycillin for three days would be as effective, in terms of clinical cure on day 4 as compared to use of oral placebo.

**3.5.** **Objectives:**

## 3.5.1.Primary Objective

To compare the proportion of children aged **2 to 59 months** presenting with non-severe pneumonia with wheezing whose respiratory rate does not fall below the age specific cut-off after three doses of nebulized salbutamol, that **achieve clinical cure** on day 4 on **3 day** of treatment with **oral amoxycillin** versus placebo

## 3.5.2.Secondary objectives

Among all cases of non-severe pneumonia with wheeze

1. To assess the proportion of children aged 2 to 59 months presenting with non-severe pneumonia with wheezing audible wheeze

2. To assess the proportion of children aged **2 to 59 months** presenting with non-severe pneumonia with wheezing who respond to up to three doses of nebulization with salbutamol**.**

Among cases of non-severe pneumonia with wheeze aged 2 to 59 months who respond to three doses of salbutamol

3. To assess the proportion of who fail therapy at day 4 of the initial successful bronchodilator therapy with inhaled salbutamol

4. To assess the proportion relapse at day 11- 14 of the initial successful bronchodilator therapy with inhaled salbutamol

5.To compare the cost of treatment of clinical failures and relapses among those treated with oral salbutamol.

6 .To assess the association of bronchodilator response with age, season, number of previous wheezing episodes, audible versus auscultatory wheeze and family history of asthma.

7.To assess the association of relapse in children who showed improvement after being treated with inhaled salbutamol, with age, respiratory syncytial virus (RSV) isolation, season, number of previous wheezing episodes, audible versus auscultatory wheeze and family history of asthma.

Among cases of non-severe pneumonia with wheeze aged 2 to 59 months who do not respond to three doses of salbutamol

8.To compare the proportion of children who are judged to be clinically cured after 3 days of treatment but who relapse within the next 11-14 days observation on 3-day treatment with oral amoxycillin versus placebo.

9.To compare the cost of treatment of clinical failures and relapses among those treated with oral amoxycillin or placebo.

10. To assess the association of clinical failure on day 4 with age, respiratory syncytial virus (RSV) isolation, season, number of previous wheezing episodes, audible versus auscultatory wheeze, family history of asthma and randomization to amoxycillin therapy.

3.5.3.Primary outcomes

- Clinical Cure: Respiratory rate below age specific cut-off (<50 bpm in infants <1 year and <40 bpm in ages 12 – 59 months) and absence of auscultatory as well as audible wheeze and no other sign of clinical failure (ie. 1,3 4) .

3.5.4.Secondary outcomes

- Audible wheeze:
- Response to nebulization: Respiratory rate below age specific cut-off (<50 bpm in infants <1 year and <40 bpm in ages 12 – 59 months) after a maximum of three doses of nebuliztion with salbutamol. Auscultarory wheeze may or may not be present. But there is no audible wheeze. That can be heard without Stesthiscop.
- Clinical failure of therapy: Clinical deterioration as indicated by the occurrence of one or more of the following:

1. Any signs of severe pneumonia or severe disease: chest in drawing, convulsions, drowsiness or inability to drink at any time.

# 2. Respiratory rate above age specific cut-off on day 4 or after that (with or without wheeze).

3. Oxygen saturation on pulse oximetry <90% on day 4 or after that.

4. Documented axillary temperature > 101 degrees Fahrenheit

In addition, children who die within the follow-up period of 14 days or are lost to follow-up on day 4 will also be considered as failed.

- Clinical relapse between day 7 -15: Signs of severe pneumonia or very severe disease among cases who were clinically cures on day 4 follow-up.

**3.6 Approach**

The study is designed to prove or disprove the hypothesis stated above. This is conditional on each study site adhering to the protocol, ensuring quality control and minimizing loss to follow-up to below 5% and is also subject to the fact that the studies continues till adequate sample size, is recruited.

**PROCEDURES AND METHODS**

**4.1 Design**

This is a prospective double blind randomized placebo controlled trial. The patients presenting in the outpatient department (OPD) of the hospitals participating in the study with cough or difficult breathing shall be seen by the medical officers on duty OPD. Children with wheeze and any sign of non-severe pneumonia shall be referred to the study physician. The study physician on receiving the patient shall assess the child for wheeze (both audible and auscultatory) along with other parameters using the WHO guidelines for ARI case management. Further assessment for enrollment in the study shall be done on patients in whom wheeze is present.

Patients will be enrolled in the study if consent to participate is obtained. After the child is enrolled in the study, baseline data will be collected. Those who fulfilled the study criteria with wheeze and fast breathing shall be given up to three doses of inhaled salbutamol by MDI or nebulizer and reassessed after each dose for fast breathing. Children in whom the fast breathing disappears will be advised bronchodilator therapy through orally at home and will be followed up and re-examined twice, on day 4, day and day 11-14. S/he would be asked to report the procedure in the health facility. The children who do not come for follow-up shall be visited at home within 24 hours of the day of follow-up. In those that fail, it is suggested that if there is history of similar illness in past the child may be given a short course of oral prednisolone 1-2 mg/kg/day in 2-3 divided doses for 3-5 days. However, if this is first episode of wheezing a course of oral antibiotic amoxicilin/ amoxiclavulinic acid combination may be given.

Children still having fast breathing after three doses of bronchodilator will be subjected to X ray film of chest for diagnosis of pneumonia. Pneumonia will be diagnosed if there are paranchymal infilterates or patch of consolidation. Children with radiological pneumonia will be treated according to the standard hospital treatment guidelines. Those without radiological pneumonia will be randomized to receive antibiotics or placebo along with oral salbutamol in doses of 0.2- 0.4 mg/kg/day in three divided doses. All children will be followed up on day 4, and 11-14 for reassessment. A child will be labelled as failed if he continues to have rapid respiration and / or wheezing. He will be treated according to practice of the hospital. In those that fail, it is suggested that if there is history of similar illness in past the child may be given a short course of oral prednisolone 1-2 mg/kg/day in 2-3 divided doses for 3-5 days. If this is first episode of wheezing a course of oral antibiotic amoxicilin/ amoxiclavulinic acid combination may be given.

If the consent is not given, they will be treated according to the standard treatment guidelines followed in the hospital.

**4.2 Stake holders plus Policy Makers**

The protocol has been planned with the inputs from the Indian Council of Medical Research (ICMR). ICMR is a national research body and with the concurrence from the government of India can assist in changing policy. The results of this study will help in refining the national policy on the management of non-severe pneumonia with wheeze. It will also give a scientific basis for or against the use of antibiotics in these cases.

The primary audience for the work is the health care providers and patients themselves in India as well as in other developing countries. The latter are also the primary stakeholders. Since the trial is being done on children, the views of the parents or guardians will be taken. The subjects will be enrolled only after obtaining informed consent. They have full rights to withdraw the child from the study or go to an alternative health care provider anytime during the trial or thereafter. Besides assessing clinical cure and failures by objective criteria, the parents will specifically be asked if they feel that their child has improved or not. Parental assessment may differ from objective clinical assessment but is important as it has a direct bearing with continuation of therapy and follow-up in the real life situation.

**4.3 Cost-effectiveness analysis**

The cost of illness and investigations in this study will be borne by the project. Therefore, it will be difficult to assess the cost-effectiveness component to the current study. If the two treatment regimens are equivalent, then the 3-day regime with placebo will be the cost-effective strategy as it is the dominant one.

However, direct and indirect medical cost of treatment of failure and/ or relapses may be different in the two arms of the study. This direct medical cost from payer’s perspective measured in the INCLEN CEU sites.

**Objectives:** The objective of cost-effectiveness analysis is to assess the incremental cost-effectiveness ratio of treatment of clinical failures and relapses in cases of non-severe pneumonia with wheeze among children between 2 – 59 months of age in 3 days amoxycillin versus placebo therapy.

**Hypothesis:**  The hypothesis of the cost-effectiveness analysis is that there is no difference in the direct medical cost of treatment of clinical failures and relapses in the two arm of the trial.

**Sample size:** The sample size has been calculated to detect a difference of Rs. 100/- with a standard deviation of Rs. 100/- for a two-tailed alpha of 0.05 and power of 0.2. We will require 20 subjects with clinical failures or relapse in each arm of the trial. Since we are assuming 17% failure rate, each site contributing 225 subjects to the trial will have adequate sample size for individual analysis for this. However, pooled data analysis will be done for all the sites participating for cost-effectiveness component of the study first.

**Design:** All such sites will have to follow-up all cases of clinical failure or relapse to assess the medical resource consumption. The unit price of all medical resources consumed will be pooled from all the participating sites and average unit price will be used for pooled analysis.

**Variables:** Data will be collected on the following medical resources utilization and the money spent on each:

|  Medicines: Types, dosage and duration of use |
| --- |
|  Investigations: Blood counts, blood gases, serum electrolytes, X- rays, ultra-sound, CT scan, MRI and any other investigation where the price of testing is > RS. 20.00 |
|  Hospitalization: Number of days in the hospital with the level of care provided |
|  Procedures: Venepuncture, thoracocentesis, Intercostal drainage, lumbar puncture, ventricular tap, all surgical procedures |
|  Consultations: Number of consultations |
|  Wages lost: Number of days the mother and/or father and any other member of the family had to stay away from work |
|  Death Human capital approach will be followed to assign a monitory value for death due to pneumonia among girls and boys and at different ages. |

**Analysis:** Analysis will be done from payers’ perspective. The price list of all the resources used will be obtained from the three hospitals in the private sector that cater to the lower middle class from each city. An average price for each resource will then be computed and used for the purpose of analysis. The units of a specific resource used will be multiplied by its average cost to get data on total expenditure on it.

Hospital care will be classified into primary, secondary and tertiary. Site-specific definitions of hospital care will be used. If site-specific definitions are not available, the hospital care will be classified into

Primary level: If the child is given oxygen by facemask and medications, by any route. Feeding is allowed or naso-gastric feeds are given.

Secondary level: If the child is in addition to above, maintained on intra-venous fluids.

Tertiary Level: If the child is kept in defined critical care unit.

Univariate analysis will be done to compare the direct medical payment on medicines, investigations, procedures, hospitalizations and consultations in the 3 days vs. 5 days amoxycillin therapy. Payment on all of the above resources will be grouped together to calculate the total medical payment for cases of clinical failures and relapses in both the arms of the trial. Student’s t test will be used for comparing the costs in both the arms of the trial.

The incremental cost-effectiveness ratio will be:

Total Cost of treatment of failures + relapses + deaths (placebo – amoxycillin arms)

# Clinical failures + relapses + deaths (placebo – amoxycillin arms)

**Implications:** Incremental cost-effectiveness ratio will provide information on the extra money spent for each case of clinical failure or relapse or death in the three days treatment with amoxycillin versus placebo. A negative ratio will favor three days therapy if both are proved to be clinically equivalent. However, a positive incremental cost-effectiveness ratio with clinical equivalence would mean that the severity of failure or relapse has to be defined and further trial with longer follow-ups may be needed before advocating no antibiotic therapy just on the basis of clinical equivalence.

**TIME-LINE**

1. Finalize protocol, submit to RSC, IRB and secure funds March. – April 2003

2. Pre-trial preparations:

Organize purchase of drugs, and randomization, printing of instruments, preparing programs for data entry and management March – May 2003

3. Constitution and 1^st^ meeting of the Data monitoring committee May 2003

4. Quality assurance workshop 26-28 Dec 2003

(For standardized training of appointed ROs at all sites in outcome assessment and data collection in Lucknow: - sending back project equipment and data collection forms)

5. Pilot (6-12 cases) Jan 2004

6. Recruitment in main study Feb 2004

7. Quality assurance site visits: June – July 2004

8. Interim analysis Jul-Aug 2004

9. 2^nd^ meeting of the data monitoring committee Aug 2004

10. Data cleaning and analysis Jul-Aug 2004

12. 3^rd^ meeting of the data monitoring committee and un-coding of the project Oct 2005

13. Report and manuscript writing workshop Dec 2005

The data monitoring committee can meet at any time on the call of the chairperson. We will budget the scheduled 1^st^, 2^nd^ and 3^rd^ meetings of the data monitoring committee and for 1 additional meeting any time.

**5. POPULATION**

**5.1 Description of sites**

The study will be carried out simultaneously in 9 hospitals namely, AIIMS (New Delhi), PGI (Chandigarh), King George’s Medical College (Lucknow), Government Medical College (Nagpur), LokManya Tilak Medical College, BMC (Mumbai), Christian Medical College (Vellore), Trivandrum Medical College (Trivandrum), Government Medical College (Bhopal), and Institute of Child Health and Hospital for Children (Chennai).

**5.2 Case definitions**

# 5.2.1 Definition of respiratory rate

The respiratory rate will be measured twice within 5 minutes or as quickly as possible while the child is quiet. The exact time that all the measurements are made will be recorded on the case report forms. The procedure for determining the valid respiratory rate will be as follows. If the first two measurements are within 5 breaths of each other, the valid rate will be the first of the to measurements. If the first two measurements differ by more than 5 breaths per minute or the second reading is below the cutoff, the respiratory rate will be measured again, as quickly as possible.

If the first and third measurements are within 5 breaths per minute of each other, the valid rate will be the first of these two measurements.

Similarly, if the second and third measurements are within 5 breaths per minute of each other the valid respiratory rate will be the first of these two measures (i.e., the second respiratory measurement).

If any two of the three measurements are not within 5 breaths per minute of each other, the respiratory rate will be measured again and the first of any two measurements within 5 breaths per minute of each other will be taken as the valid respiratory rate used for decision making.

All measurements will be recorded for later analysis.

5.2.2. Non-Severe Pneumonia

Cough and fast breathing, defined as respiratory rate of > 50 per minute (children 2 to 11 months) or >40 per minute in children 12 – 59 months of age.

5.2.3.Severe pneumonia

Cough and/or fast breathing with lower chest wall retraction and nasal flaring, grunting respiration.

**5.2.4 Wheeze**

Look to see when the child is breathing OUT. A child with wheezing makes a soft musical noise during expiration or shows signs that breathing OUT is difficult. Wheeze refers to a noise heard either with or without stethoscope.

Audible wheeze - Listen for the wheeze by holding your ear near the child’s mouth, since the noise may be difficult to hear. The narrowing of the air passages in the lungs causes wheezing. The breathing out takes longer than normal and requires effort. Sometimes so little air moves that there is little to no noise. Look to see if the breathing out phase requires great effort, and is longer than normal. If the child is wheezing, ask the mother if her child has had a previous episode of wheezing within the last year. A child with recurrent wheeze has had more than one episode of wheeze in a 12-month period.

Auscultatory wheeze **-** When the air moves in and out of narrow air passages the above mentioned soft musical sound can be heard with the stethoscope even in the absence of an audible wheeze. Sometimes a health worker can confuse a conducted sound with wheeze. Conducted sounds usually are heard equally well during both phases of respiration while wheeze is best heard when the child breathes out. Clear the secretions in the nostril of the child by using a cotton wick. The conducted sound noise would either disappear or will become less in intensity.

**5.2.5 Lower chest wall indrawing**

Lower chest wall indrawing or chest indrawing must be persistent to record “Yes”. When looking for indrawing health workers should ask the mother to expose the lower part of the chest wall and look for drawing in of the ribs and/or sternum with inspiration. If they cannot see this or they are unsure, they should record “No” for this question. Care must be taken to distinguish this from out ward movement of a distended abdomen with inspiration. Lower chest wall indrawing is apparent when the lower third of the chest wall moves in on inspiration. It may be useful to compare the motion of the chest wall with a fixed point behind the baby’s chest. In most cases sub-costal indrawing is indistinguishable from lower chest wall indrawing. Occasionally indrawing occurs without visible inward movement of the bony structure, record this as sub-costal indrawing only.

**5.2.6 Abnormally sleepy or difficult to wake**

An abnormally sleepy child is drowsy most of the time when the child should be awake and alert. This sick child will not look at the mother or watch your face when you talk. This child may stare blankly and may not appear to see. Ask the mother if the child has seemed unusually sleepy or difficult to wake. Look to see if the child wakens when the mother talks, or when you clap your hands. A child who is difficult to wake may continue to sleep even with the mother’s voice or a loud clap. Even a very young baby, who sleeps a lot, should waken naturally with these disturbances, or when the mother begins to undress the child.

5.2.7 Unable to drink

Inability to drink at all in a child aged 2-59 months.

# 5.2.8 Stopped feeding well

# A young infant (up to 2 months) stops feeding well, when s/he takes less than half of the usual amount of milk.

**5.2.9 Stridor**

Look when the child is breathing IN. Stridor is a harsh noise made when the child breathes IN. Listen for stridor by holding your ear near child’s mouth, since the noise may be difficult to hear. Stridor occurs when there is narrowing of the larynx, trachea, or swelling of the epiglottis, which interferes with air entering the lungs. These conditions are also called croup. Sometimes one hears a wet noise if the nose is blocked. Clear the nose, and listen again. Often a child who is not very ill will have stridor only when the child is crying or upset, so be sure to look and listen for stridor when the child is calm.

5.2.10 Fever or low body temperature

A temperature of 38^o^C or 100.4 ^o^F or above is regarded as fever. Below 35.5^o^C or 96^o^F is an abnormally low temperature, called hypothermia. Use a low reading thermometer that can measure temperatures below 35^o^C or 95^o^F. Temperature will either be measured in axilla or rectum, which will be recorded on the appropriate case report forms.

5.2.11. Asthma

Asthma is defined as a diffuse, obstructive airway disease with

- hyperactivity of the airways to a variety of stimuli,
- a high degree of reversibility of the obstructive process, which may occur either spontaneously or as a result of treatment.

For the purpose of the project, any child with a history of more than three episodes of documented wheeze in a year will be regarded as asthmatic.

**5.2.12. Allergic rhinitis**

Recurrent rhinitis is chronic or recurrent clear nasal discharge usually associated with sneezing, conjuctival itching and injection.

5.2.13. Eczema

Eczema may be defined as a particular type of skin reaction which includes exudation, lichenification and pruritis. It may be acute or chronic.

- Acute eczema is characterized by erythema, weeping, oozing, and micro vesicles in epidermis of skin.
- Chronic eczematous lesions are thickened, dry and scaly with coarse skin markings and altered pigmentation.

**5.2.14.Urticarial Rash**

This signifies vary degree of appearance of 1-3 mm multiple wheals and flare with angioedema. These lesions are evanescent resolving within minutes to hours only to appear anywhere else.

**5. 2.15. Biomass Fuels(Wood, Coal, Kerosene, Dung cakes)**

**5.3 Inclusion criteria**

1. Age - 2-59 months
2. Audible/auscultatory wheeze
3. Respiratory rate above age-specific cut off point (Table 1)

**5.4 Exclusion criteria**

1. Presence of danger sign

- 2-59 months old

-unable to drink

-convulsions

-abnormally sleepy or difficult to wake

-stridor in calm child

1. If the child has received adequate documented antibiotic treatment in the past 48 hours i.e.

- Cotrimoxazole 4 doses,
- Ampicillin 8 doses,
- Amoxycillin 6 doses,
- Chloramphenicol 6 doses,
- Erythromycin 4 doses,
- Clarithromycin 4 doses,
- Cefixime 2 doses,
- Azithromycin 2 doses,
- Inj. Benzyl penicillin 8 doses,
- Inj. Procaine penicillin 2 doses)
- Any other suitable antibiotic, in the previous 48 hours.

1. Complicating acute non-pulmonary or chronic illness e.g., known cardiac disease, severe dehydration, known renal failure, chronic pulmonary disease (e.g. cystic fibrosis, known bronchiectasis, bronchopulmonary dysplasia, cough > 30 days, pulmonary tuberculosis), severe anaemia, known G6PD deficiency. These children will have an associated illness leading to investigations and treatment not included in the protocol of the study that can influence the response to treatment.
2. Children that cannot be followed up e.g. living outside the municipal limits of the city.
3. Children whose parents or guardians refuse to give consent.
4. Previous enrollment in the present study.

**5.5 Sample size**

**5.5.1. For equivalence trial**

The proposed trial is a randomized double blind placebo controlled trial running simultaneously in 9 centers. The trial will compare the proportion of children with non-severe pneumonia and wheeze who are do not improve or are clinical failures at the end of 3 days in the 3 amoxycillin therapy versus placebo.

The criteria used for sample size estimation are as follows:

1. The anticipated proportion of children aged 2 to 59 months presenting with cough and fast breathing, with wheezing (non-severe pneumonia) who do not improve by the end of three days on 3 day course of treatment with oral amoxycillin versus placebo, respectively, or who are clinical failures any time during the 3 day period is 17% in both the arms.
2. The maximum difference from the true estimate which can be accepted is > 5% and < 10%, with a power of 90% and alpha level of 0.05, using a one tailed t-test.
3. The effect of 5% loss to follow-up and multiple sites (10 study sites) have been identified has been adjusted for in the calculation.
4. The sample size then for the estimate being off by 1%, 2%, 3%, 5%, 7.5% and 10% from the true value is given in the table below

Formula for equivalence sample size

(Z β+ Zα^2^ (p1*q1+p2*q2)

N =-----------------------------------------

(p1- p2 - (δ*)2

Where δ* is the difference which the clinicians allow (recommend) to conclude that the two treatments are equal.

We would say that the two mode of therapies are equal if the failure rate in the new regimen is not more than 17% (difference is within 5%). This 5% is δ*. We permit this difference (effect is more in the new arm) , because there are other benefits such as less cost, less side effect etc.

If we assume that the p1 and p2 are equal, then the above formula becomes

(Z α+ Zα^2^ ) * 2*p*q

n =-----------------------------------------

( - δ*)2

The alpha then becomes 0.03 from 0.05 and keeping the power of 90%, the required sample size for various δ* is as follows:

Delta Star (%) Sample Size Sample Size Adjusted

2 5283 5853

3 2348 2602

4 1321 1464

4.5 1044 1157

5 846 937

**Thus, each arm will have 950 subjects. Each site contributes 225 patients over 18 months.**

**5.6 Randomization**

This is a double blind randomized trial. Block randomization will be done in Dept. of Pharmacology, Upgraded KGMC, Lucknow, which is not the coordinating center for the trial. Blocks will be generated in mixed batches. Dr. Sanjay Khattri , who is not the biostatistician responsible for the design and analysis of the study, nor is on the data monitoring committee will be responsible for random allocation (Randomization Officer-in-Charge). The medicines will be then placed in serially numbered envelopes by the pharmacy according to the intervention type determined by the pharmacy. Two hundred and twenty five random numbers will be generated in blocks of varying lengths for each of the nine sites.

Medicines for the 3 days will be placed in colored envelopes. Eleven doses (9 required doses and 2 extra) of either amoxycillin or placebo for the first 3 days will be provided for a 20 KG child in the green envelope. .

The envelopes for each site will be serially labeled and along with their contents will be mailed to the coordinating center, Lucknow. For serial labeling the sites will be listed alphabetically according to the city and within city by the name of the institution, if required. The list is as follows:

Name of city and institution Serial numbers allocated

**Bhopal 1-225**

**Chandigarh 226-450**

**Chennai 451-675**

**Lucknow 676-900**

**Mumbai 901-1125**

**Nagpur 1126-1350**

**New Delhi 1351-1575**

**Trivandrum 1576-1800**

**Vellore 1801-2025**

Required supplies for a center will be send at the beginning of the study. The supplies will be stored in local areas in a locked cupboard. After an envelope has been used, the subject and site identification number, date of randomization and the name of the project staff in whose presence the envelope was opened will be written on the envelop in appropriate space. The white envelope will be pinned with the data sheets of the subject and mailed to the coordinating center along with it.

The face of the envelope will have space for the following writing of the data:

ISCAP-II Trial

- Serial Number: will be written ( 1-1975)
- Site identification number: [___]

(1=Bhopal, 2=Chandigarh, 3 = Chennai, 4 = Lucknow, 5=Mumbai, 6 = Nagpur, 7= New Delhi, 8=Trivandrum, 9= Vellore)

- Subject identification number: [___][___][___]

(From 1 upto 225 per site)

- Date of randomization: [___][___]/[___][___]/[___][___][___][___]

M M D D Y Y Y Y

The envelope will also have the following instructions for use in local language:

ISCAP – II Trial

Instructions for use

- Envelope must have been opened in front of the project staff
- First dose from the envelope must have been given under direct supervision of the project staff
- Give one dose three times a day
- If a child vomits within half hour of a particular dose, give another dose.
- Note that after giving 9 doses from the envelope some medicine will remain unused if your child weights less than 20 KG. Leave those doses unused and bring them back on the next follow-up visit.
- If your child develops any of the following symptoms please contact your site physician and bring the child to the hospital immediately:

Lower chest retraction Inability to drink,

Convulsions Abnormally sleepy or difficult to wake

Diarrhea Skin rash

Vomiting Nausea

The medicines will be provided in scored dispersible tablets. Each tablets has 125 mg of AMX. They will be used after dissolving in 5 ml of clean water. The medicines will be given according to the weight of the child as follows:

- 4 – 6 KG ½ tablet thrice a day
- 7 - 10 KG 1 tablet thrice a day
- 11 – 15 KG 1 ½ tablet thrice a day
- 16 – 20 KG 2 tablets thrice a day

Accordingly to weight of the child remove extra tablets before handing over the envelop.

**5.7.** **Enrollment**

Patients will be recruited from those coming to out patients’ department or emergency rooms in large secondary as well as tertiary care hospitals in India. While each site will evolve its own methodology of recruiting as many patients as possible, they will all follow the standard recruitment procedure. The procedure involves a two stage screening prior to initiation of the consent procedure and randomization. The first screening is a verbal screening by the project nurse and the second screening is a standardized-recorded screening by the project medical officer.

The study nurse will verbally screen all the patients between the ages of 2-59 months of by asking the following questions:

1. What is the age of your child?
2. Is your child suffering from any of these today: cough, fast breathing, and difficult breathing?

Details will be kept on the number of children screened by the nurse. It is expected that the site investigators will ask her/him to screen as many patients as possible. Subjects who are of relevant age and have any of the three symptoms from cough, fast breathing and difficult breathing will be sent to the medical research officer for detailed and recorded screening. The parents will be requested to take the child to pre-designated area for further detailed examination by the project medical research officer (M-RO). The M-RO will examine the child for the presence of inclusion criteria and exclusion criteria. Those who are potentially eligible for the study (inclusion criteria present and all exclusion criteria absent) will then be auscultated for the presence or absence of wheeze. All other children will be sent to the regular out patients’ clinic or emergency room for standard management as per the hospital protocol.

Axillary temperature will be measured on all the potentially eligible children. Axillary temperature > 38 degrees C, as measured by the digital electronic thermometer until the instrument signals that the reading is complete and confirmed by second recording, will receive paracetemol syrup in the dose of 10-15 mg/Kg/dose. The subject management of potentially eligible children will be different for those with and without wheeze. For those with wheeze at enrollment, respiratory rate assessment will be done after relevant nebulization, as described below, since this therapy take more than 30 minutes, by which time the antipyretic would have had its effect.

- Those *with wheeze* will be nebulized three times with salbutamol (ref. IP) at intervals of 20 minutes. Respiratory rate will be counted after 20 minutes of the third nebulization according the described protocol. If the respiratory rate fulfills the inclusion criteria will be subjected to baseline screening, in which all cases of severe pneumonia who were missed in the prior screening are excluded. Those not excluded at this stage are now invited to participate in the informed parental consent procedure prior to enrollment in the study and randomization..
- The enrollment procedure after presentation to a specific site is as follows:
- Three stage triage for candidate selection

Stage 1: *Preliminary verbal screening* by the project nurse for presence of inclusion criteria

Stage 2: *Detailed* s*creening* by the project medical officer for presence of inclusion criteria and absence of all exclusion criteria

Stage 3: *Baseline assessment* by the project medical officer to re-confirm presence of inclusion criteria and absence of all exclusion criteria.

- Informed written consent of potentially eligible subjects
- Enrollment of consenter for either discriptive study ro RCT followed by randomization for latter randomization

A record will be maintained of the potentially eligible children coming to for the detailed screening in all the participating sites.

**For a site to stay in the study a minimum of 5 subjects must be randomised in any month period and day 4 and day 11-14 follow-up must be more than 95%. In addition, the site must follow the clinical and microbiological parts of the protocol stringently. To complete the study within the stipulated time frame, each site is required to make all efforts to enroll at least 4 and a maximum of 8 cases per week into the study. This would give a sample size of about 225. Care must be taken to recruit cases in all twelve months of a year to represent all seasons.**

**5.7 Consent**

The purpose of the study will be explained to parents and oral informed consent to participate obtained. A proposed consent form is attached (**Form C**). The parents or legal guardians of the children eligible to enter the study will be fully informed about the study. The site investigator from all the institutions involved in the study will obtain the freely given, written consent of the parents or legal guardian for the child to participate in the study. The content of the explanation provided to the parents or legal guardians of the children is described in the attached consent form (see Form **C1+C2)** If the parent or legal guardian is not literate, a thumbprint may be substituted for signature, duly witnessed by somebody in addition to the person requesting consent.

**6. VARIABLES AND INTERVENTIONS**

**6.1 Variables**

Children referred to the investigator or the project medical officer will be subjected to detailed screening, using **Form A**, to evaluate whether they fulfill each of the inclusion criteria and have none of the exclusion criteria. Children who are ineligible for the study will be treated according to the standard procedures at each site. Those who fulfill the inclusion criteria and have no exclusion criteria will be subjected to baseline assessment. The purpose of the baseline assessment is to re-confirm the eligibility of the subject for participation in the study and to exclude those not eligible prior to randomization. Parents of children who are judged to be eligible for participation after the baseline assessment by the medical officer will be requested to participate in the informed written consent procedure prior to randomization.

OUTCOME ASSESSMENT

Baseline assessment

The baseline assessment, using **Form B**, will be performed as quickly as possible after the detailed screening. This should be done within 4 hours after presentation to the site. The baseline assessment includes re-evaluating to ensure that no signs of severe pneumonia or very severe disease are present. Data collected at baseline include:

1. Name, address, detailed description of location and other contact information
2. Details of date of birth correct to the year of birth and nearest month and age in months.
3. History of present illness (duration of symptoms etc.) including history of asthma and allergic disorders in the family and child. This includes asthma, rhinitis, hay fever or eczema. History of exposure to indoor pollutants will also be elicited.
4. Medical history including presence of known or suspected immunodeficiency (congenital or acquired)- malignancy being treated with chemotherapy or radiotherapy, steroid treatment, suspected HIV infection as manifested by a history of Pneumocystis carnii pneumonia, lymphoid interstitial pneumonia, esophageal candidiasis, or the occurrence of recurrent bacterial or viral infections, miliary/extrapulmonary/cavitatory tuberculosis, herpes zoster, systemic cytomegalovirus infection, neurological dysfunction, oral thrush, failure to thrive, continuous or intermittent fever of > 1 month duration, persistent or intermittent diarrhea of > 14 days, generalized lymphadenopathy and generalized dermatitis in patient with history of exposure (parents with known HIV, AID, blood or blood product transfusion, non-sterile scarification/ear piercing or circumcision in high prevalence HIV area.
5. Weight, length (in children under 24 months) and height (in children > 24 months) obtained by standard procedures (copy from Form A)
6. Valid respiratory rates (copy form Form A)
7. Presence or absence of wheeze
8. Heart rate
9. Axillary temperature using a digital electronic thermometer

Children who are no longer eligible for the study at the completion of baseline evaluation (e.g. because signs of severe pneumonia or very severe disease have appeared) will be discontinued from the study and receive usual care as clinically indicated. All the data will be recorded. Eligible children will be randomized to 3-day amoxycillin therapy versus placebo group and nasopharyngeal aspirate for estimating RSV by Beckton Dickenson kit will be taken.

The study personnel will explain and demonstrate to the mother how to give the treatment at home at recruitment and at all subsequent follow-up visits.

Assessment on day 4 (73 – 96 hours)

The “zero” will be the time of enrollment. The study personnel will call the child for the fourth day follow-up visit and will examine the child. The family will be paid the travel allowance according to the local conditions. The M-RO will review the key variables. Those children with wheeze will be provided with bronchodilator therapy and advised how to administer it. Mothers will also be advised to give paracetemol in the same dose if the temperature is more than 38 degrees C, if they have facilities to measure it; else they would be advised to administer paracetemol if the child feels hot touch. All the medications for the current illness of the subject enrolled in the trial will be provided free of cost from the project funds.

All the mothers will be taught to recognize the signs of worsening illness according to those outlined in the WHO ARI management chart. They will be advised to the hospital if any of the signs develop at any time before the scheduled visit. They will also be given 24 hours contact telephone number. The mothers will be instructed to bring back the envelope with remaining medicines at the first follow-up visit on day 4. The on the first follow-up visit at day 4. The mother will be instructed to keep the wrappers, bottles and prescriptions of all medicines purchased or provided from non-project sources and administered to the at all times, specifically between day 4 and the last follow-up visit between days 11-14. The following observation will be made and recorded in case report forms:

- - 1. Presence or absence of wheeze
    2. Valid respiratory rate
    3. Valid oxygen saturation by pulse oxymetry
    4. Heart rate
    5. Axillary temperature by digital thermometer
    6. Signs of severe pneumonia - chest indrawing, cyanosis, nasal flaring, grunting
    7. Signs of very severe pneumonia – convulsion, drowsiness, stridor, inability to drink
    8. Number of doses of various medicines taken

Project nurse will visit the homes of the patients with in the same day if the child is not brought to the hospital.

**Day 4 Assessment**

On the first follow-up visit at day 4 (73 – 96 hours) an assessment of clinical failure will be made. Clinical failure will be judged by the presence of one or more of the following:

1. ***Clinical deterioration as indicated by the occurrence of one or more of the following: chest in drawing, convulsions, drowsiness or inability to drink at any time.***

# *2. Respiratory rate above age specific cut-off on day 4 or after that.*

# *Oxygen saturation on pulse oximetry <90% on day 4 or after that.*

1. ***Documented temp > 101 0F***.

Presence of clinical failure or severe adverse reaction will require change in the antibiotic therapy for the child. The change in therapy will be determined by the site investigator based on the local hospital guidelines and condition of the child but it is suggested that amoxycillin and first-generation oral cephalosporin can be considered as alternative modes of therapy. In cases of clinical failures and severe adverse reactions, the decision to perform any investigations, including x-ray chest, will be taken by the site investigator.

**Assessment on 11-14**

The M-RO will call back all the enrolled subjects on day 11-14. The occurrence of clinical relapse will be assessed among cases that were clinically cured on day 4. Project field worker will visit the homes of children who do not keep this appointment within the time frame .

**6.3 Study treatment**

Salbutamol will be used as the bronchodilator drug of choice. It will be administered by metered dose inhaler (MDI) either using spacer device in older children and spacer device with a face mask in younger children or nebulizer in the hospital.

**6.3.1 Salbutamol by MDI**

Children under five years old lack the coordination for using a metered-dose inhaler by them. They often cannot use the mouthpiece or breathe in when the inhaler is pressed. To overcome these problems, a spacer device shall be used. This will allow the salbutamol to be vaporized into an enclosed space as given in Figure 2 below. The child breathes in through a hole. For younger children a facemask should be used which is placed over their nose and mouth.

6.3.2 Standardisation of salbutamol dosage

To overcome the electrostatic discharges created in the plastic volume spacer initially 4-6 actuations will be used to coat the inside surface of spacer device. At the time of screening four actuations each will be used for up to three times at 0, 15 and 30 minutes as necessary. This procedure will be carried out under the supervision of the study physician. After enrollment the patient shall be advised to use two actuations of inhaled salbutamol per dose. Details of dosage schedule are given in Table 2.

## Table 2. Dosage schedule for salbutamol inhalations:

| **Drug** | **Age** | **Dose*** |
| --- | --- | --- |
| At screening |  |  |
| Salbutamol aerosol | 2 - 59 months | 400 μ gm per dose up to three times 15 minutes apart |
| At home |  |  |
| Syrup Salbutamol ( 2mg/5ml) | 2 - 12 months  13 – 59 months | ½ teaspoon thrice a day  1 teaspoon thrice a day |
| **Assessment at follow-up**  (if signs of pneumonia present) |  |  |
| Salbutamol aerosol | 2 - 59 months | 400 μ gm per dose up to three times 15 minutes apart |

* One actuation of MDI contains 100 μ gm

**6.2.3 Procedure for the use of MDI with spacer device:**

Demonstrate the use of MDI and the spacer to the child and mother/guardian as follows:

1. Before the use of spacer device make sure that valve is functioning. Hold the spacer device in your hand and shake the spacer gently. The noise produced by the valve shows that the valve is functional.
2. Remove the cap of the inhaler (MDI), hold the MDI in your hand and shake it a couple of times. Insert the MDI into the opening for MDI as shown in the figure.
3. Place the valve end of the spacer in your mouth. Close your lips tightly around the opening. Press down the canister of the MDI firmly. A mist will escape into the spacer device. Now breathe deeply in and out through your mouth at least 5 times so that the aerosol reaches your lungs.
4. Now ask the mother/guardian and patient to use the MDI and spacer on the child, as demonstrated.
5. Check carefully all the steps of and patient the procedure and correct any deficiencies in the method.
6. Ask the mother/guardian to repeat the procedure till you are satisfied with the technique.
7. For small children who cannot use the MDI by themselves a spacer device with a facemask shall be used and its use shall be demonstrated to the caretaker.

**Figure 2. Meter Dose Inhaler (MDI) plus spacer**


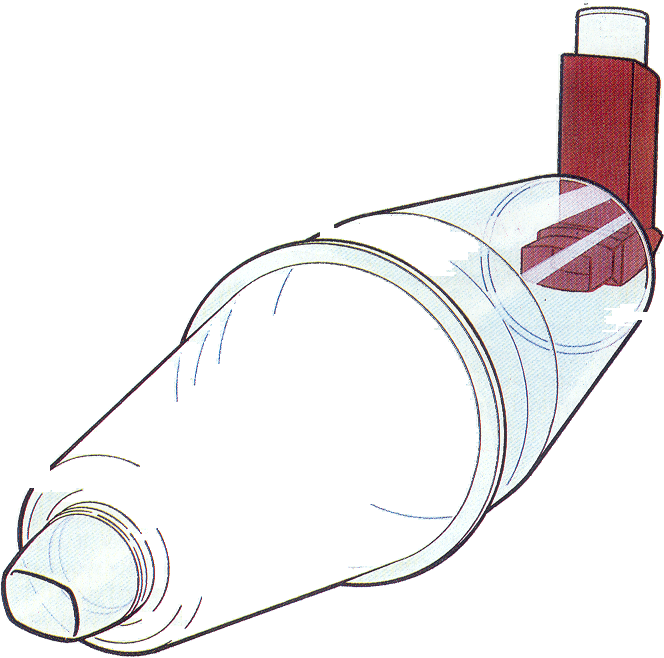


**Nebulization dose 0.03ml/kg (o.15 mg/Kg)**

**6.4 Change of therapy**

The indications for the change in antibiotic therapy for children randomized in the study will be as follows:

1. Clinical failures on day 4 assessment or any time before

2. Adverse drug reaction any time in the three days of treatment

The site investigator will decide regarding the change of antibiotics and investigations, based on the hospital policies and the condition of the child. It is suggested that the antibiotic may be changed to oral amoxycillin (40 mg/kg every 8 hours) or first generation cephalosporin (25 mg/kg every 8 hours) for children with clinical signs of pneumonia. A child having signs of severe pneumonia or very severe disease or not clinically improving after 48 hours of oral changed antibiotic therapy may be considered for parental therapy. All children with clinical findings of severe pneumonia or very severe disease may be referred for admission and appropriate treatment. All children, referred or not, whose treatment is changed will be re-evaluated after 48-72 hours and until cured. At any stage during the observation period, any child who deteriorates clinically will be referred for admission.

**6.5 Non-compliance**

Compliance for amoxycillin and *salbutamol* will be evaluated at each follow-up by counting the number of doses taken. A child will be judged to be non-compliant to amoxycillin if less than 7 out of 9 doses have been given from the envelop for the three days of therapy. *Again, if the child takes less than 7 out of 9 doses of salbutamol for three days of therapy will be judged as non-compliant to salbutamol.*Those who have not been compliant, but are not clinicalfailures and have not experienced severe adverse reaction will be asked to follow the study protocol.

**6.6 General Supportive Care**

1. Fever: Where facilities are available, patients with axillary temperature > 38 degrees C, as measured by the digital electronic thermometer until the instrument signals that the reading is complete and confirmed by second recording, will receive paracetemol syrup in the dose of 10-15 mg/Kg/dose as needed, with a minimum 6 hours difference between doses. Otherwise, mother will be advised to treat the child with paracetamol if the body is hot to touch. Tepid or cold water sponging is discouraged because it increases oxygen consumption, carbon-dioxide production and is uncomfortable.
2. Feeding: Mothers will be encouraged to breast feed their child. Meals will be offered according to usual practice at each site. Children will be offered normal diet during illness.
3. Hydration: We do not expect dehydration to be a significant problem in the study children, but if present will be managed according to standard WHO rehydration protocol.
4. Bronchodilators: All wheezing children will receive nebulized salbutamol, 0.5 ml of a 5 mg/ml solution in 2 ml normal saline, nebulized over a period of 5 minutes. Since the children will be going home after randomization, oral salbutamol syrup (2mg/5ml) is a dose of ½ teaspoon per dose for children less than 1 year and 1 teaspoon per dose to children between 1-4 years of age will be given.
5. Nasal secretions: Saline nose drops will be used if the nose is blocked with thick and dry mucus.
6. Oxygen, and drugs like epinephrine, aminophylline and corticosteroids will not be used in any of these patients unless the patients have been classified as treatment failures. The patient’s physician will determine the use of these treatments.
7. Anti-histamines and cough syrup will not be used in any of these children as they have no beneficial effect, make children drowsy and may interfere with accuracy of the other assessment.
8. Other antibiotics- No other antibiotic (oral or injectable) are allowed while the child is enrolled in the study. If other antibiotics are considered necessary for treatment of the child, he/she will be discontinued from the protocol, classified as a clinical failure and treated as clinically indicated.

**6.7 Discontinuation of study**

It is the responsibility of the site investigator to maintain the child in the study, provided it is safe to do so. A child may be discontinued from the study for any of the following reasons, that must be documented on the appropriate case report form:

- 1. Clinical failure
  2. Occurrence of a serious adverse reaction
  3. Parents or guardians withdraw support
  4. Protocol is not followed

Clinical Care of Children who are discontinued from the study

Children classified as clinical failures will receive antibiotic therapy as directed by their physician. The physician may also obtain additional chest radiographs, blood counts, C-reactive protein, blood cultures and other laboratory tests to aid patient management according to clinical judgment and the usual practice of the participating site.

Children who experience serious adverse events will also be discontinued from the therapy. Antibiotics other than penicillin related drugs would be given to these patients, as well as bronchodilators, paracetamol, and other therapy as appropriate.

Parents who refuse to have their children continue to participate in the trial any time will be advised about the general care of the child and how to recognize increasing severity of illness. Children will be treated according to the clinical condition of the child at withdrawal.

Procedures at discontinuation of enrollment

The site investigator is responsible for completion of all appropriate case report forms up to the time that the child is discontinued from the study.

**6.8 Study instruments**

There are 11study forms that will be completed by all the sites for each subject recruited. There are 2 extra forms for unscheduled visit of the enrolled case. For centers participating in the cost-effectiveness component of the study additional 6 forms have to be completed for cases who are clinical failures or have relapsed.

Table 2: Various study instruments for the ISCAP Trial

| S.No | Form number | Purpose |
| --- | --- | --- |
| 1. | Form A | Detailed Screening Form |
| 2. | Form B | Base Line Assessment Form |
| 3. | Form C | Informed Parental Consent Form (in local Language) |
| 4. | Form D | Day 4 assessment of clinical failure Form (Scheduled visit) |
| 5. | Form E | Day 1-3 assessment Form (Unscheduled visit) |
| 6. | Form F | Day 6 assessment of cure Form (Scheduled visit) |
| 7. | Form G | Day 13 – 15 assessment for relapse Form (Scheduled visit) |
| 8. | Form H | Day 7 – 12 assessment Form (Unscheduled visit) |
| 9. | Form I | Adverse reaction reporting Form |
| 10. | Form J | Death Reporting Form |
| 11. | Form K1 | Cost-effectiveness analysis: Medicines used Form |
| 12. | Form K 2 | Cost-effectiveness analysis: Investigations done Form |
| 13. | Form K 3 | Cost-effectiveness analysis: Procedures done Form |
| 14. | Form K 4 | Cost-effectiveness analysis: Hospitalization Form |
| 15. | Form K 5 | Cost-effectiveness analysis: Consultations Done Form |
| 16. | Form K 6 | Cost-effectiveness analysis: Workdays lost Form |
| 17. | Form L | Summary Assessment Form |

**Form C1 (page 1 of 1)**

ISCAP II

Informed Parental Consent Form

[___]/[___][___][___][___]/[___]/ [___][___][___][___]

(site/serial/study type: 1= descriptive, 2=RCT/study no. for study type 1 and random number for study type2)

**Purpose of the study:**

Children with wheeze and fast breathing, whose respiratory reduces to normal after bronchodilator therapy do not require antibiotic treatment. Oral bronchodilators are sufficient. The purpose of the this study is to assess natural course of such episodes.

###### Study procedures

Your child has been diagnosed to have wheeze which can safely be treated at home. We invite you to participate in this study. If you are willing to have your child participate, S/he will get oral bronchodilator for 3 days. Medicine will be as syrup and has to be given thrice a day. You are required to bring your child for follow-up on day 4 and 11-14 days after enrollment.

###### Risks from the study

No serious risks are anticipated from participation. However it is possible that some children will not recover completely. Your child will be followed closely and additional treatment given if required. Oral salbutamol is a commonly used drug for treatment of wheeze and has a good safety profile but side effects are rarely reported.

###### Benefits from the study

Results of this study will give information on natural course of children with wheeze. It will provide information for management of such children and identify future areas of research.

The benefits to your child will be that he/she will be provided close medical follow up free of cost by a separate physician and will not be required to wait. All medications for this episode of illness will be provided free of cost.

**Form C1 (page 2 of 3)**

Informed Parental Consent Form

Unique identifier:

[___]/[___][___][___][___]/[___]/ [___][___][___][___]

(site/serial/study type: 1= descriptive, 2=RCT/study no. for study type 1 and random number for study type2)

Complications

We do not anticipate any serious complications during the study. However, complications may occasionally arise during the course of the study, either due to the disease process or due to treatment. The doctors at no cost to you will carry out treatment of such complications. No financial compensation will be provided for such complications.

Compensation

You will be compensated for travel for follow up visit. There will be no other financial compensation for participation in the study.

Confidentiality

All information collected in this study will be kept strictly confidential, except as may be required by law. Your child or family will not be identified by name if the results of the study are published.

Rights of the participants

Participation in the study is voluntary. Refusal to participate will not influence care of your child in this hospital in any way. Though we would like all study participants to complete the study, you are free to withdraw from the study at time during the course of the study. If at any time during the course of the study, you have any questions or concerns related to the study, you may contact the following doctor:

[Enter name, address and telephone number of site PI]

Alternatives to participation in the study

Your child will be seen by one of the doctors in the clinic and provided treatment according to the regular treatment protocol of the hospital.

Consent

I have had the study explained to me and have read the contents of this form/had the contents of this form read to me. I have been given the opportunity to ask questions and have them answered to my satisfaction. I am willing for my child to be enrolled in the study.

**Form C1 (page 3 of 3)**

Informed Parental Consent Form

Name of the subject:

Unique identifier:

[___]/[___][___][___][___]/[___]/ [___][___][___][___]

(site/serial/study type: 1= descriptive, 2=RCT/study no. for study type 1 and random number for study type2)

Signature of parent/guardian ______________________________ Date: _________

Name: ____________________________

Relationship to subject:_____________________________

Signature of the Investigator: _____________________________ Date: __________

Name of the investigator: ____________________________________

Signature of witness: ___________________________________ Date: __________

Name of witness: ____________________________________

Name of the Subject: ____________________________________

Father's Name : ____________________________________

Address: __________________________________________________

__________________________________________________

__________________________________________________

**Form C2 (page 1 of 3)**

ISCAP II

Informed Parental Consent Form

Unique identifier: [___]/[___][___][___][___]/[___]/ [___][___][___][___]

(site/serial/study type: 1= descriptive, 2=RCT/study no. for study type 1 and random number for study type2)

## Purpose of the study

Children with a chest infection (pneumonia) with wheeze require treatment with a medicine called bronchodilator. Such children may not require antibiotic but improve with only bronchodilator therapy. In this study, we propose to compare treatment of three-day amoxycillin and bronchodilator with three- day placebo and bronchodilator.

*Study procedures*

Your child has been diagnosed to have pneumonia with wheeze, which is not severe and can safely be treated at home. We invite you to enroll your child in this study. If you are willing for your child to be enrolled in the study, he/she will be given an antibiotic (amoxycillin) or placebo with an oral bronchodilator for 3 days. Your child will have an equal chance to be in either group. Neither you nor the doctor will know the contents of the tablets, which look identical, though this information will be available to your doctor, if necessary. Medicine will be supplied in the form of dispersible tablets, which will have to be given three times daily. You will be required to bring the child for follow up on 4 and 11-14 days after commencing treatment. A specimen will be collected from the nose for laboratory testing at enrollment. A urine sample will also be taken.

*Risks from the study*

No serious risks are anticipated in this study. We are assuming that recovery will be equal following 3 days of treatment. However, it is possible that some children will not recover completely. Your child will be closely followed up and additional treatment administered, if necessary. The antibiotic amoxycillin and bronchodilator or salbutamol are one of the commonly used treatments for pneumonia. They have a good safety profile but side effects are rarely reported.

Collection of specimens from the nose will cause mild temporary discomfort to the child, but most children tolerate this well.

*Benefits from the study*

The results of this study will benefit society by providing information that may justify the use of shorter courses of antibiotics that will result in considerable cost savings and may prevent bacteria (germs) from developing antibiotic resistance.

The benefits to your child will be that he/she will be provided close medical follow up free of cost by a separate physician and will not be required to wait. All medications for this episode of illness will be provided free of cost.

**Form C2 (page 2 of 3)**

Informed Parental Consent Form

Unique identifier:

[___]/[___][___][___][___]/[___]/ [___][___][___][___]

(site/serial/study type: 1= descriptive, 2=RCT/study no. for study type 1 and random number for study type2)

Complications

We do not anticipate any serious complications during the study. However, complications may occasionally arise during the course of the study, either due to the disease process or due to treatment. The doctors at no cost to you will carry out treatment of such complications. No financial compensation will be provided for such complications.

Compensation

You will be compensated for travel for follow up visit. There will be no other financial compensation for participation in the study.

Confidentiality

All information collected in this study will be kept strictly confidential, except as may be required by law. Your child or family will not be identified by name if the results of the study are published.

Rights of the participants

Participation in the study is voluntary. Refusal to participate will not influence care of your child in this hospital in any way. Though we would like all study participants to complete the study, you are free to withdraw from the study at time during the course of the study. If at any time during the course of the study, you have any questions or concerns related to the study, you may contact the following doctor:

[Enter name, address and telephone number of site PI]

Alternatives to participation in the study

Your child will be seen by one of the doctors in the clinic and provided treatment according to the regular treatment protocol of the hospital.

Consent

I have had the study explained to me and have read the contents of this form/had the contents of this form read to me. I have been given the opportunity to ask questions and have them answered to my satisfaction. I am willing for my child to be enrolled in the study.

**Form C2 (page 3 of 3)**

Informed Parental Consent Form

Name of the subject:

Unique identifier:

[___]/[___][___][___][___]/[___]/ [___][___][___][___]

(site/serial/study type: 1= descriptive, 2=RCT/study no. for study type 1 and random number for study type2)

Signature of parent/guardian ______________________________ Date: _________

Name: ____________________________

Relationship to subject: _____________________________

Signature of the Investigator: _____________________________ Date: __________

Name of the investigator: ____________________________________

Signature of witness: __________________________________ Date: __________

Name of witness: ___________________________________

Name of the Subject: ________________________________

Father's Name : ____________________________________

Address: __________________________________________

__________________________________________________

__________________________________________________

**6.10.Quality Control**

Patients will be recruited from these coming to OPD in large secondary as well as tertiary case hospitals in India (in the cities of Lucknow, Chandigarh, Delhi, Bombay, Trivandrum, Vellore, Chennai, Bhopal and Nagpur). A project orientation workshop was held in KG’s Medical College, Lucknow from 9-11October 2002. All the site investigators met to discuss the project in-depth. A consensus protocol was decided upon following deliberations on the draft protocol circulated. The final project will be submitted to the RSC of IndiaClen and ethics clearance committee of AIIMS and Upgraded King George’s Medical College, Lucknow and thereafter to the IndiaClen IRB.

Once the project has been cleared by the IRB, each site will recruit research officers and a centralized orientation workshop will be organized for them to ensure standardization and quality control.

**Proposed schedule of training**

Day 1: Management of ARI, detection of wheeze, Training for the use of MDIs

Day 2: Mock enrollment of the patients and filling of CRFs

Day 3: Further enrollment of the patients and follow-up at home and hospital

Day 4: Discussion and feedback from study physicians on CRFs.

Thereafter, a pilot case recruitment will be done in the next 4 weeks. It is expected that the team will become well conversant with the procedural details. Any time there is a change of the medical research officer for the project, the site investigator will train the replacement and inform the co-ordinating center. An external reviewer will visit all the sites after the initiation of the study for quality assurance.

During this visit, the first few patients will be recruited in the study. The activities, which will be performed during each visit, will include the following:

Meeting the site investigator and members of the study staff

Review of study procedures, use of data forms and related documents

Observation of clinical personnel carrying out specific procedures

Physical walk-through of certain procedures (e.g., series of examinations needed to determine patient eligibility or steps followed to obtain informed consent)

Conversation with key support personnel to assess their practice with regard to data collection

Inspection of storage facilities (drugs) and study facilities

A random subset of records from each site will be evaluated for quality control. Investigators will be asked to bring all medical records (and results of laboratory tests, etc.) for the selected subjects to the data analysis workshop. Information in the medical records will be compared with the data on the case report form to assess completeness and accuracy of reported data.

**7. DATA ANALYSIS AND MANAGEMENT**

**7.1 Data management plan**

All the sites will sent a fortnightly report on case recruitment to the co-ordinating center either by fax or e-mail on 1^st^ and 15^th^ of each month. All patient forms, except the written and informed consent form, will also be sent to the co-ordinating center once a month (1^st^ of each month) for all cases with completed follow-up. However, adverse reaction and death forms will be sent to the co-ordinating center as soon as possible within 24 hours of information of their occurrence to the co-ordinating center by fax.

Co-ordinating Center

Data entry into dBase or ACCESS based data management system will include

- Double data entry with online edits (range and logic checks)
- Status variables that permit constant tracking of data fields, form and participant status
- Edit reports, form and participant status reports
- Password control of access to system and control of access to particular system functions (such as making changes or corrections to data)
- Automatic audit trail capability that tracks changes to data, monitors use of system features and keeps track of user log-ins

Data are entered into a transaction database. Edit reports are run from this database. When errors and edits have been resolved (with audit trails that monitor the nature of the error as well as the original and all corrected values), the validated data are posted to a master database. All databases are backed up on a daily basis for security.

**7.2 Data Analysis Plan**

The data will be double entered in the co-ordinating center, Lucknow. The cleaned data will be analyzed. Univariate analysis will be done for

Comparison of pre-randomization characteristics across sites and across the two arms of the trial.

1.Compare the number of potentially eligible children reporting at various centers

2.Compare the recruitment rates in all the 9 participating sites

#### 3. Compare the patient characteristics across all the sites and across the two arms of the trial. This includes age, sex, weight, height, weight-for-age z-scores, height-for-age z-scores and the proportion of children underweight and stunted

4.Compare the symptoms along with their duration across the nine sites and across the two arms of the trial.

5. Compare the RSV isolation rate at the initiation of therapy across all sites and then across both the arms.

Comparison of loss to follow-up at each site and across both the arms

1. Number of children lost to follow-up on days 1,2,3,4,5 and 10-12 at each site and then in both the arms will be compared.
2. Reasons for loss to follow-up, if any, will be ascertained.

**Comparison of outcomes**

Univariate Analysis

1. Primary outcome, that is the proportion of children who are clinically improved on day 4 will be compared across both the arms. Sub-group analysis will also be done by site
2. Secondary outcomes will be compared across both arms

3. Association of the primary outcome with other pre-randomization variables

For univariate analysis, chi square test will be used for categorical data.

Student’s t test for continuous data with 2-group comparison and analysis of variance for more than 2 group comparison. Delta star of 5% will be taken into consideration during analysis. Using a chi square or test distribution, a p value of <0.05 will be considered statistically significant. With the exception of primary outcome comparison when a single tailed p value will be used, a two-tailed p value will be used for all other comparisons. All results will be reported with their 95% confidence interval.

Multivariable Analysis

1. Logistic regression analysis will be done, if required, to predict the primary outcome, using as potential predictors the randomization group, presence or absence of wheeze at enrollment and any other variable that has univariate association with the primary outcome and is clinically meaningful and improves the goodness of fit of the final model.

**7.3 Bias in data collection**

This is a double blind placebo controlled trial. Block randomization will be done. This will reduce the chances of bias in data collection. Study data will be collected on case report forms (CRFs). The sites will Xerox the case report forms and sent the original ones to data co-ordinating center in Lucknow. Once a fortnight site investigator or his/her designate will send a progress report to the progress co-ordinating center in New Delhi either by fax or e-mail. Study specific forms will be provided to each center. Each month the site investigator or his/her designate will be responsible for visually editing the CRFs for each child who has completed the study or has been withdrawn from it. All missing data should be identified and every attempt should be made to complete the missing data. The site investigator or designate will be responsible for mailing the CRFs to the co-ordinating center monthly.

**7.4. Study management at site**

Each site will be responsible for setting up an information system to keep track of all patients screened and enrolled and a filing system to keep all study records – case history records, study protocol or related documentation and drug distribution records. The site investigator is responsible for the completeness and accuracy of the study materials.

Case History Records

These include the study case report forms (CRFs) that will contain information that documents the child’s ability to participate in the study (including a copy of a signed consent form) and information from tests and examinations. Whenever possible copies of supporting documentation of the information contained in the CRF should be kept with each patient’s case history records. The supporting documentation may include records of physical examination, progress notes, laboratory reports, X-rays, consultations, patient’s condition during and after clinical investigation, concomitant therapy etc. All information in the case history records should be attributable to a specific individual. Since the CRFs must not contain the patient’s name, there must be a unique link between the ID number of the CRF and the patent’s name. Each child’s case history record will be evaluated to verify the validity and completeness of the data on the CRF when a study monitor visits the study site.

All corrections to the CRFs must be made without obscuring the original entry. The revised entry should be inserted and the person making the correction should sign and date the correction. Only authorized study personnel may complete or correct case report forms.

Study protocol and related documentation

All study related documents including the study protocol, manuals of operation, all correspondence sent to or received from the study monitor, materials used for obtaining informed consent, protocol modifications and records for the Institutional Review Board approval and all documents will be evaluated to ensure that study documentation is complete and current when a study monitor visits the site.

Records retention

Retention of accurate and complete records is essential to establish the validity and completeness of the study. All records must be retained for 5 years after the data set is frozen.

**7.5. Project monitoring & reviews**

The Site PI must agree with and sign the protocol and confirm in writing that he or she has read, understands and will work according to the protocol and Good Clinical Practice. The Site PI is responsible for making sure that the protocol is strictly followed and should not make any changes to the study unless necessary to eliminate an apparent immediate hazard or damage to a study subject.

Any deviations from the study protocol including but not limited to inappropriate enrollment of a study subject, administration of the wrong treatment, missed doses of study treatment, missed observation points etc. should be reported to the coordinating centre and each site’s IRB. The report should include a plan to rectify any problems at the site that may have caused the protocol deviation.

We have planned for an interim analysis after approximately one-third of the data has been collected. Annual project progress will be assessed with out unblinding or outcomes assessment for presenting the data to the IRB. A data monitoring committee will be constituted which will meet at pre-specified intervals. The data monitoring committee will be given the same progress report at the IRB. Information of all adverse reactions will be given to the data monitoring committee as well as the IRB. The decision for stopping the trial prematurely will rest on the data monitoring committee.

**7.6 Limitations of the study**

This study will not assess the etiological causes of non-severe ARI since it is a drug efficacy trial. Therefore, the study will not be able to identify if there are specific etiological types of non-severe ARI, which are not benefited by 3 days amoxycillin therapy. Also since all children with wheeze, irrespective of history of asthma, will be enrolled it may be possible that the asthmatics versus non-asthmatics may behave differently. In this situation the sample size of the study will not be adequate for either.

**7.7 FDA Investigational new device or Investigational Device Exemption**

Neither amoxycillin nor salbutamol is a new drug. Both have received FDA approval for use in non-severe ARI in children. Therefore, we apply for investigational device exemption.

**8. HANDLING OF UNEXPECTED OR ADVERSE EVENTS**

**8.1 Adverse effects of salbutamol**

These include tremors, tenseness, headache, peripheral vasodilatation and a compensatory small increase in heart rate and tachycardia. Hypersensitivity, muscle cramps and hyperactivity in children are rare. Potentially serious hypokalaemia may result from β 2-agonist therapy. Cardiac arrhythmias (including atrial fibrillation, supraventricular tachycardia and extra systoles), although rare may occur in some patients.

## Interactions of salbutamol with other drugs

## Salbutamol and non-selective beta-blockers such as propanolol should not usually be prescribed together.

**8.2 Adverse effects of amoxycillin**

Response to new or unexpected findings and changes in the study environment

Antibiotics such as amoxycillin are generally well tolerated and are rarely associated with serious or life threatening adverse reactions (possibly or probably related to drug therapy). The most common side effects reported include:

- Allergic – skin rash, hives, itching or wheezing
- Digestive – nausea, vomiting, diarrhea
- Metabolic – moderate or transient increase in liver function tests – transaminases
- Hematological - anemia, leucopenia and irreversible thrombocytopenia

Rare side effects include anaphylactic shock, Stevens- Johnson syndrome and pseudomembranous colitis.

Precautions for use

Amoxycillin dose should be adjusted for renal failure according to creatinine clearance. When administering in high doses of amoxycillin, sufficient liquids should be administered to ensure an adequate diuresis.

Allergic reactions may require amoxycillin to be stopped and an alternative (non-beta lactam) antibiotic to be used. Anaphylaxis and other serious adverse events are more common after parentral administration, but may occur with oral administration of amoxycillin. A careful history of problems associated with prior antibiotic use should be obtained from the child’s parent or guardian.

## Drug Interactions

Allopurinol should not be administered while a child is taking amoxycillin because of the increased risk of skin rash. Large doses of amoxycillin can interfere with assays of blood glucose, urine glucose and total serum proteins.

Carcinogenesis, mutagenesis and impairment of fertility

Long term studies to evaluate carcinogenesis and mutagenic potential of amoxycillin in animals have not been done. Studies in rats and mice treated with doses of amoxycillin up to 10 times the usual human dose have not resulted in any impairment of fertility or adverse effects on the fetus.

Overdosage

Possible manifestations of amoxycillin overdosage include neuropsychiatric, renal or digestive manifestations. Amoxycillin can be eliminated through hemodialysis.

**8.3** **Identifying, managing and reporting adverse events.**

The site investigator will carefully monitor each child for adverse events. If an adverse event occurs, the site investigator will assess its duration, seriousness, intensity and relationship to the duration of study medication (salbutamol). The principal investigator will use their judgement about whether to continue the child in the study or to discontinue enrollment. The event and its treatment will be reported on appropriate CRF.

### Definitions of adverse events:

*Adverse events* – any undesired experience occurring in a child during a study, whether or not the event is considered to be related to the therapy under investigation.

*Serious adverse event* – any fatal, life threatening, disabling event or event that results in hospitalization or prolongation of hospitalization.

*Unexpected adverse event* – an experience not previously reported (nature, severity or incident in the currently provided drug data sheets or package inserts).

All adverse events including those observed by the study personnel or problems, complaints, signs or symptoms volunteered by the child or their parent or guardian and diagnoses must be recorded on the serious and non-serious case report forms provided, regardless of whether they are associated with the study treatment. All adverse events may be associated with the concomitant illnesses, reactions to medications or progression of other diseases (excluding pneumonia). Whenever possible, the adverse events should be evaluated and reported as a diagnosis rather than individual symptoms and signs. If a definitive diagnosis is not possible, individual signs and symptoms should be reported.

### Relationship to the study drug

The relationship of the study drug to an adverse event is classified as none, remote, possible, probable or not assessable. The decision is clinical, based on all available information at the time of the CRF. Factors to consider include:

- Temporal relationship between the drug administration and the occurrence of the event. The event should occur after the drug is administered if it is considered related to drug administration. The length of time from drug exposure to event will be evaluated in the clinical context of the event.
- Recovery of discontinuation (de-challenge), or occurrence on re-introduction (re-challenge).
- Underlying, concomitant, intercurrent illness. The event should be evaluated in the context of the natural history of the disease being treated and any other diseases that the patient may have.
- Concomitant medication or treatment. Other medications that the patient is taking should be examined to determine whether any are known to cause event in question.
- Known response pattern for this class of drug.
- Exposure to physical or mental stresses. Any exposure to stress might cause changes in the recipient and provide a logical better explanation for the event.

Severity of an adverse event

The intensity (severity) of the event should be classified as:

- Mild- transient, not interfering with the child’s activities;
- Moderate- causes sufficient discomfort to interfere with the child’s activities;
- Severe- incapacitating and prevents normal activities.

If the intensity changes over time, the maximum intensity should be recorded.

### Reporting of Serious Adverse Events

Since salbutamol and amoxycillin are widely used, is not investigational at any study site and is recommended for treatment of children who have wheeze, it will not be necessary to report the occurrence of serious adverse events to the coordinating center within 24 hours of the investigator knowing about the event. The site investigator will be responsible for obtaining full information on the event and completing the appropriate case report forms. These case report forms will be batched and sent monthly to the coordinating center. The study principal investigator should send a copy of the adverse event data to the coordinating centre as soon as possible. The coordinating centre will summarize the adverse event information and send a report to the data safety-monitoring committee (DSMC).

**9. COMMITTEES**

9.1 Data Safety monitoring Committee (DMC)

Even though amoxycillin is widely use a data safety monitoring board will be constituted. This will have as members renowned individuals with varied experiences, specifically in the areas of randomized trials and epidemiological study design and child health and acute respiratory infections and will be neither associated with study design, implementation, data collection, funding or donations for the work. The suggested speciality of persons for the data monitoring committee is as follows:

-Biostatistian of International standing preferably

-Pediatrician of International standing with knowledge of management of acute respiratory preferably diseases

-Physician with knowledge of methodology of randomized trials, of International standing and preferably with origin in the Indian subcontinent.

The project investigators have suggested three names in each of the three above-mentioned categories as potential members of the data monitoring committee. Once the project has been cleared by the IRB, the study principal investigator and the study co-ordinator will contact the potential members of the data monitoring committee sequentially. The member listed next in order will be contacted if the previous member has either refused to participate or has not responded within the stipulated time of the 15 days. The DMC will meet in March-April 2003 and one of the three will be made the chairperson of the data monitoring committee. The rules and regulations of the data monitoring committee will be formulated at its first meeting and circulated to the investigators and the IRB. The project co-ordinator will send information to the Chairperson data monitoring committee from time to time as decided. Any serious adverse reaction, which includes death during the duration of the study, will be informed to the data monitoring committee immediately. The data monitoring committee will order for the opening of codes at the end of the trial or any time in between, if required.

The names of the suggested persons of eminence are as follows:

**Biostatistian:**

1. Dr. NC Saxena, ICMR, New Delhi
2. Dr. Richard Peto, CTSU, Oxford, UK
3. Dr. Kant Bagdiwala, UNC, Chapel Hill, USA

**Pediatrician**:

1. Dr. Indu Wakhlu, Ex-head, department of Pediatrics, KGMC, Lucknow
2. Prof. Thomas Cherian, WHO, Geneva
3. Dr. Oliviere Fontaine, WHO, Geneva

**Physician with knowledge of methodology of randomized trials:**

1. Prof. J.N. Pandey, Prof. And Head of department of Medicine, AIIMS, New Delhi and Co-ordinator CEU, AIIMS and President IndiaClen
2. Dr. JayPrakash Muliyil, Dean, CMC, Vellore
3. Dr. Nick White, Mahidol University, Bangkok, Thailand

**9.2 Steering Committee**

A Steering Committee will compose of the Principal Investigator from each of the site participating in this multicentre study; one representative from each of the sponsors will be constituted to assure the leadership of the study. The Steering Committee will assumes the responsibility for general design and conduct of the trial, including preparation of essential study documents such as manual of operations, data forms, and treatment protocols. It will review data collection practices and procedures, as summarized in performance monitoring reports, from visits to participating clinics and other means, to identify and correct remediable deficiencies. It will also consider and adopt changes in study procedures as desirable and essential during the course of the trial. Appointment and disappointment of the subcommittees needed for the execution of the trial also forms a part of its functions. It will make decision on resource allocations and on priorities for meeting competing demands in the trial and will review progress of the study in achieving its main goals and take steps required to enhance likelihood of success in achieving them.

It will review and implement recommendations for a treatment protocol change, such as termination of a treatment because of lack of efficacy.

***Decisions within the Committee will be reached by consensus.***

**10. DISSEMINATION, NOTIFICATION AND REPORTING**

The use of data and publication of the results derived from this multicentre study was discussed during the proposal development workshop, and the following recommendations were developed by consensus.

All data derived from this multicentre study will be the property of the investigators and the sponsors. The Steering Committee will set up rules for access to the data by groups outside the sites participating in the multicentre study, and when the data will become available in the public domain.

When the data set is completed and “frozen” it will be made available to each country team. This “frozen” data set will be used for any subsequent site-specific analysis.

The principal paper(s) on the primary and secondary outcomes emerging from the multicentre study will be published under a corporate authorship.

The names of the scientists who have participated in the Proposal Development and Data Analysis Workshops will be listed at the end of the paper in alphabetical order and their specific contribution to the study will be mentioned. Members of the coordinating groups (ARCH Project, Boston University and CAH, WHO) will be named there.

Sites participating in the multicentre study will then be listed by alphabetical order and the selection of authors within each country will be the responsibility of the site Principal Investigator. It is understood that authorship, within each site, will be offered to a limited number of scientists who have made substantive contribution to the study. It is expected that the number of authors listed per site will not exceed four but the final determination resides with the site Principal Investigator. Within each site, the order of the author’s names will be the responsibility of the site Principal Investigator. Each site will provide a list of persons to be mentioned in the Acknowledgments, under an agreed guidance for the type of support that must be acknowledged.

Each site team is encouraged to produce scientific manuscripts and technical reports based on their site-specific data. They agree to postpone publication of site-specific data until after the main paper has been accepted for publication. It is expected that acceptance for publication will occur within 18 months after completion of the data analysis workshop. If publication of the main paper takes more than 18 months, this moratorium will have to be re-considered by the Steering Committee. After the main paper has been accepted for publication or after the Steering Committee has agreed to it, the site teams are free to publish results as appropriate, and are encouraged to acknowledge that the site-specific data was collected as part of a larger multicentre study.

Additional research questions may be investigated using the combined data set. Any investigator from the “core study group” can identify additional research question/paper ideas. However, before undertaking such additional analyses the investigator will submit a concept paper on the proposed analysis for review by the Steering Committee. In papers emerging from additional analyses, the final responsibility for identifying and listing the authors resides with the principal author. However, the multicentre study group will be acknowledged and mentioned at the end of the authorship line.

Scientists are encouraged to present the findings at scientific conferences and meetings. When scientists are invited to international or regional meetings, the Steering Committee should be informed of their intent to present the data, and its approval of the content of the presentation will be required.

The sponsors as well as the institutions participating in the multicentre study may disclose data derived from the study to national or foreign drug regulatory authorities, even prior to acceptance for publication of the principal paper.

**11. ETHICAL CLEARANCES**

The local institutional ethics committee will clear the study first. Thereafter it will be submitted to the IndiaClen- USAID IRB for clearance since the US-India Mission (Infectious disease initiative) money will fund the work.

**12. BIBLIOGRAPHY**

1. Sachdev HPS, Mahajan SC, Garg A. Improving antibiotic and bronchodilator rescription in children presenting with difficult breathing: experience from an urban hospital in India. Indian Pediatr 2001; 38: 827-838.

1. .Selwyn BJ. The epidemiology of acute respiratory tract infection in young children: Comparison of findings from several develop-ing countries. Coordinated Data Group of BOSTID Researchers. Rev Inf Dis 1990; 12: S870-S888.
2. . Glezen P, Denny FW. Epidemiology of acute lower respiratory disease in children. N Engl J Med 1973; 288: 498-505.
3. El Radhi AS, Barry W, Patel S. Association of fever and severe clinical course in bronchio-litis. Arch Dis Child 1999; 81: 231-234.
4. Weber MW, Dackour R, Usen S, Schneider G, Adegbola RA, Cane P, et al. The clinical spec-trum of respiratory syncytial virus disease in the Gambia. Pediatr Infect Dis 1998; 17: 224-230.
5. Prahl P, Peterson NT, Homsieth A. Beta 2-agonists for the treatment of wheezy bron-chitis. Ann Allergy 1986; 57: 439-441.
6. Alari AJ, Lewander WJ, Dennehy P, Seifer R, Mansell AL. The efficacy of nebulized metaproterenol in wheezing infants and young children. Am J Dis Child 1992; 146: 412-418.
7. Bentur L, Canny GJ, Shields MD, Karem E, Schuh S, Reisman JJ, et al. Controlled trial of nebulized albuterol in children younger than 2 years of age with acute asthma. Pediatrics 1992; 89: 133-137.
8. Mallol J, Barrueto L, Girardi G, Munoz R, Puppo H, Ulloa V, et al. Use of nebulized bronchodilators in infants under 1 year of age: Analysis of four forms of therapy. Pediatr Pulmonol 1987; 3: 298-303.
9. Chavasse RJ, Bastian-Lee Y, Richter H, Hilliard T, Seddon P. Inhaled salbutamol for wheezy infants: A randomized controlled trial. Arch Dis Child 2000; 82: 370-375.
10. Cates C J, Rowe BH, Bara A. Holding chambers versus nebulisers for beta-agonist treatment of acute asthma. The Cochrane Library, 2: 2002.
11. Mandelberg A, Tsehod S, Houri S, Gilad E, Morag B, Priel IE. Is nebulized aerosol treatment necessary in the pediatric emer-gency department? Chest 2000; 117: 1309-1313.
12. Kenyon C, Thorsson L, Borgstrom L, Newman S. The effects of static charge in spacer devices on glucocorticosteroid aerosol deposition in the asthmatic patient. Eur Respir J 1998; 11: 606-610.
13. Wildhaber JH, Devadason SG, Eber E, Hayden MJ, Everard ML, Summers QA, et al. Effect of electrostatic change, flow, delay and multiple actuations on the in vitro delivery of salbutamol from different small volume spacers for infants. Thorax 1996; 51: 985- 998.
14. Zar H, Brown G, Donson H, Braithwaite N, Mann M, Weinberg E. Homemade spacers for bronchodilator therapy in children with acute asthma: A randomized trial. Lancet 1999; 54: 979-982.

16. Awasthi S. Clinical response to two days oral amoxycillin in children with non-severe pneumonia. Indian Pediatrics 2000;37:301-306

17.Schrag S, Pena C, Fernandez J, Sanchez J, Gomez V, Peraz E, Feris J, Besser R. Effect of short course, high dose of amoxycillin therapy of resistant pneumococcal carriage. JAMA 2001;286:49-56

18.Clinical efficacy of three days versus five days of oral amoxycillin for the treatment of childhood pneumonia: a multicentric double- blind trial. Lancet July 2002: 1-7

19.Francisco AD, Chakraborty J. Adherence to co-trimoxazole treatment for the acute lower respiratory tract infections in rural Bangladeshi children. Annals of Tropical Pediatrics 1998;17-21

1. GCP Guidelines

Appendix II

ISCAP I RESULTS

Table I Showing the equivalence of two treatment types

| Intervention type | A | B |  |
| --- | --- | --- | --- |
| Total Recruitment | 1095 | 1093 |  |
| Cure N , % | 888 , 81.1 | 915 , 83.7 | χ_2_^2^ = 2.87, p=.238 |
| Failures N , % | 164 , 15.0 | 137 , 12.5 |
| Relapses N , % | 43 , 3.9 | 41 , 3.8 |
| Loss to Follow-up (day 6) N , % | 57 , 5.2 | 62 , 5.7 | χ_1_^2^ =0.23 , p=0.63 |
| CE study Recruitments N , % | 137 , 12.5 | 129 , 11.8 | χ_1_^2^ =0.258 , p=0.611 |
| RSV + N , % | 252 , 23.0 | 261 , 23.9 | χ_1_^2^ =0.228 , p=0.633 |
| Strep Pneum Isolates & N , % | 648 , 59.2 | 628 , 57.5 | χ_1_^2^ =0.67 , p=0.41 |

Table 2 Showing the resistance of isolated Streptococcus Pneumonia and Haemophilus influenzae to co-trimoxazole stratified by treatment type as well as by wheeze

|  | Treatment A | | Treatment B | | P Value, Chi^2^ | |  |
| --- | --- | --- | --- | --- | --- | --- | --- |
|  | Wh+ N , % | Wh- N , % | Wh+ N , % | Wh- N , % | Treatment A | Treatment B |  |
| # (%) Resistant SP-Time() | 26 , 61.9 | 227 , 67.2 | 32 , 71.1 | 220 , 65.5 | p=0.49  χ_1_^2^=0.46 | p=0.45  χ_1_^2^=0.56 |  |
| # (%) Resistant HI-Time() | 12 , 57.1 | 106 , 54.1 | 18 , 64.3 | 115 , 60.5 | p=0.79  χ_1_^2^=0.07 | p=0.7  χ_1_^2^=0.145 |  |
| # (%) Resistant SP-Time 2 | 15 , 88.2 | 91 , 64.1 | 9 , 64.3 | 102, 79.7 | p=0.046  χ_1_^2^=3.98 | p=0.19  χ_1_^2^=1.75 |  |
| # (%) Resistant HI-Time 2 | 13 , 76.5 | 61 , 54.5 | 8 , 72.7 | 56 , 58.9 | p=0.087  χ_1_^2^=2.92 | p=0.38  χ_1_^2^=0.78 |  |
| P value time 0 vs 2 for SP  (Chi Sq Test) | χ_1_^2^=3.96  p=0.048 | χ_1_^2^=0.42  p=0..5 | χ_1_^2^=0.23  p=0.63 | χ_1_^2^=8.81  p=0.003 |  | | |
| P value time 0 vs 2 for HI  (Chi Sq Test) | χ_1_^2^=1.56  p=0.21 | χ_1_^2^=0.0001  p=0.95 | χ_1_^2^=0.25  p=0.62 | χ_1_^2^=0.07  p=0.8 |

**Appendix III**

**Lohd`fr lfgr vuqefr i=**

(ISCAP – II)

Form C2 Page (1 of 3)

Unique Id: [___]/[___][___][___][___]/[___]/ [___][___][___][___]

(site/serial/study type: 1= descriptive, 2=RCT/study no. for study type 1 and random number for study type2)

**v/;;u dk mís';%**

ftl cPps dks QsQM+s dh lwtu vkSj Nkrh dh ?kqj?kqjkgV (Wheeze)

gks tkrh gS] mUgs ,slh nok dh vko';drk gksrh gS ftldks ,UVhck;ksfVd vkSj czkUdksMkbysVlZ (bronchodilators) dgrs gSaA ftu cPpksa dks Nkrh dh ?kqj?kqjkgV (wheeze) Hkh gksrh gS] mUgs ,UVhck;fVd dh vko';drk ugha gks ldrh gS] dsoy czkUdksMkbysVlZ (bronchodilators) ls Bhd gks tkrh gSA bl v/;;u esa ge yksx rhu fnu ,UVhck;ksfVd ,eksfDllhyhu (amoxycilline) + czkUdksMkbysVlZ (bronchodilators) dh rqyuk rhu fnu fuf"Ø; nok (placebo)+ czkUdksMkbysVlZ (bronchodilators) ls dj jgs gSaA

**v/;;u ds rjhds%**

vkids cPps dks QsQM+s dh lk/kkj.k lwtu gS ¼ftlesa [krjk ugha gS o ftls fueksfu;k Hkh dgrs gS½ ,sls esa vkids cPps dk bykt lqjf{kr :i ls ?kj esa gks ldrk gSA ge vkids cPps dks bl v/;;u esa Hkkx ysus ds fy, vkidks vkeaf=r djrs gSA ;fn vki cPps dks bl v/;;u esa Hkkx gSA fnykuk pkgr@pkgrs gS rks mls ,UVhck;ksfVd ,eksfDllhyhu + czkUdksMkbysVlZ (bronchodilators) ;k fuf"Ø; nok + czkUdksMkbysVlZ (bronchodilators) igys rhu fnu nh tk,xhA vkids cPps dks 3 fnu ds bykt ;k 5 fnu ds bykt ds lewg esa lfEefyr gksus dh ,d lh lEHkkouk gSA igys 3 fnu nksuks lewg ds cPpksa dks ,UVhck;ksfVd feysxhA pkSFks vkSj ikapos fnu tks cPps 3 fnu dh nok lewg esa lfEefyr gS] dks ,slh xksyh nh tk,xh ftlesa ,UVhck;ksfVd ugha gSA u rks vkidks u vkids fpfdRld dks irk gksxk fd dkSu lh xksyh esa D;k gS D;ksafd nksuksa ns[kus esa ,d lh yxrh gS ijUrq vko';drk iM+us ij vkids fpfdRld dks bldh lwpuk ns nh tk,xhA nok ikuh esa /kqy tkus okyh xksfy;ksa ds :i esa nh tk,xh ftls vkidks vkids cPps dks izfrfnu 3 ckj nsuh iM+sxhA vkidks cPps dh tkap ds fy, pkSFks vkSj X;kjg ls pkSng fnu ds chp ykuk iM+sxkA cPps dh ukd ls :bZ ds Qk;s ls tkap ds fy, uewuk fudkyk tk,xk v/;;u ds izFke fnuA

**v/;;u ls lEHkkfor [krjs%**

bl v/;;u esa Hkkx ysus ls xEHkhj lEHkkfor [krjs ugha gS geyksx vuqeku djrs gSa fd 3 fnu ;k 5 fnu ds bykt esa leku :i ls cPps Bhd gksxsA ijUrq ;g Hkh gks ldrk gS fd dqN

ISCAP- II

Form C2 Page (2 of 3)

Unique ID: [___]/[___][___][___][___]/[___]/ [___][___][___][___]

(site/serial/study type: 1= descriptive, 2=RCT/study no. for study type 1 and random number for study type2)

cPps iwjh rjg Bhd u gksA vkids cPps dk lw{e :i ls fujh{k.k djrs jgsaxs vkSj ;fn vko';drk gqbZ rks vfrfjDr bykt Hkh fn;k tk,xkA ,UVhck;ksfVd ,ekDfllhyhu fueksfu;k esa lk/kkj.k nh tkus okyh ,d nok gSA lqj{kk dh n`f"V ls ;g ,d vPNh nok gS] ij vlk/kkj.kr% blls Hkh vfrfjDr nok ls lEcfU/kr y{k.k gks ldrs gSA ukd dh tkap ls cPps dks {kf.kd rfud rdyhQ gks ldrh gS ij vf/kd cPps bls vPNh rjg lgu djrs gSaA

**v/;;u ls ykHk%**

bl v/;;u ds Qy ls lkeqnkf;d ykHk gks ldrk gS ftlls de fnu ds fy, ,UVhck;ksfVd dk bykt fn;k tk,xk ftlls vkfFkZd cpr gksxh vkSj chekjh ds thok.kq ls ,UVhck;ksfVd ds fo:) izfrjks/k dh 'kfDr mRiUu djus dh {kerk esa {kfr igq¡psxhA

vkids cPps dks ;g ykHk gksxk fd mls lw{e fujh{k.k fu%'kqYd ,oa fpfdRld }kjk iznku dh tk,xh blds fy, mls :duk ugha iM+sxk bl chekjh ds bykt ds fy, lHkh nok fu%'kqYd nh tk,xhA

**ijs'kkfu;ka%**

bl v/;;u esa geyksx dksbZ Hkh xEHkhj ijs'kkuh gksus dh lEHkkouk ugha dj jgs gSaA ijUrq dHkh&2 v/;;u ds nkSjku dqN&dqN ijs'kkfu;ka gks ldrh gSa ;k rks chekjh ds dkj.k ;k mlds bykt lsA bu lHkh ijs'kkfu;ksa dh fpfdRlk MkDVj yksx fu%'kqYd djsaxaaasA bl ijs'kkfu;ksa ds fy, dksbZ Hkh vkfFkZd Hkqxrku ugha fd;k tk,xkA

**Hkqxrku%**

vkidks vkus tkus dk fdjk;k fn;k tk,xk] tc vki izR;sd tkap ds fy, cPps dks yk,xsaA blds vfrfjDr dksbZ Hkh vkSj vkfFkZd Hkqxrku bl v/;;u esa Hkkx ysus ds fy, ugha fd;k tk,xkA

**xksiuh;rk%**

bl v/;;u esa ,d= dh x;h lwpuk xksiuh; j[kh tk,xh vkSj dsoy mruh gh vkSj jk; gh nh tk,xh tc dkuwu vfuok;Z djsxk vkids cPps dk uke ;k ifjokj dk uke v/;;u ds fu"d"kZ dks izdkf'kr djrs le; ugha izdkf'kr fd;k tk,xkA

#### **Hkkxhnkjh ds vf/kdkj%**

bl v/;;u esa Hkkx viuh Lor% bPNk ls fy;k tkrk gSA blls Hkkx ysus ds fy, euk djus ls vkids cPps ds lEHkky esa vLirky dh vksj ls dksbZ Hkh ifjorZu ugh vk,xk ;|fi ge vkis{kk djrs gS fd vki iwjs v/;;u esa Hkkx ys] vki vius cPps dks fdlh Hkh le; v/;;u

ISCAP- II

Form C2 Page (2 of 3)

Unique ID: [___]/[___][___][___][___]/[___]/ [___][___][___][___]

(site/serial/study type: 1= descriptive, 2=RCT/study no. for study type 1 and random number for study type2)

ls gVkus ds fy, iw.kZr% Lora= gS v/;;u ds fdlh Hkh le; ;fn v/;;u ls lEcfU/kr dksbZ Hkh iz'u gks] rks vki fuEufyf[kr MkDVj dks lEidZ dj ldrs gSaA

Mk0 'kSyh voLFkh]

cky foHkkx] ds0 th0 esfMdy dkyst] y[kuÅ] Qksu u0 2257329

v/;;u esa Hkkx u ysus ij vU; mipkj% vkids cPps dks dksbZ ,d MkDVj vLirky esa ns[k ysxk vkSj vLirky dh lk/kkj.k i)fr ds vuqlkj bykt djsxkA

**Lohd`fr%**

eq>s bl v/;;u ds ckjsa es le>k fn;k x;k gS vkSj eSus bl Lohd`fr i= dks i<+k gS i<+ok;k gSA eq>s iz'u iwNus dk volj fn;k x;k gS vkSj lHkh iz'uksa dk larks"ktud mRrj feyk gSA eS vius cPps dks bl v/;;u esa Hkkx ysus ds fy, Lohd`fr nsrk gw¡@nsrh gw¡A

ekrk&firk@ vfHkHkkod ds gLrk{kj % ---------------------- frfFk --------------------

uke% ---------------------------------------------------------------------------------------------

cPps ls lEcU/k% ------------------------------------------------------------------------------

tkapdrkZ dk gLrk{kj% ------------------------------------------- frfFk -------------------

tkapdrkZ dk uke% ----------------------------------------------------------------------------

xokg dk gLrk{kj% ---------------------------------

xokg dk uke% --------------------------------------

cPps dk uke % ------------------------------------------------------------------------------------------

firk dk uke % ------------------------------------------------------------------------------------------

irk % ------------------------------------------------------------------------------------------

-----------------------------------------------------------------------------------------------------------

Qksu uEcj % ----------------------------------------------------------------
